# Identification of tissue-specific and cold-responsive lncRNAs in *Medicago truncatula* by high-throughput RNA sequencing

**DOI:** 10.1186/s12870-020-2301-1

**Published:** 2020-03-06

**Authors:** Mingui Zhao, Tianzuo Wang, Tianyang Sun, Xiaoxi Yu, Rui Tian, Wen-Hao Zhang

**Affiliations:** 1 State Key Laboratory of Vegetation and Environmental Change, Institute of Botany, the Chinese Academy of Sciences, Beijing, 100093 People’s Republic of China; 2 Research Network of Global Change Biology, Beijing Institutes of Life Science, the Chinese Academy of Sciences, Beijing, 100101 People’s Republic of China; 3 University of Chinese Academy of Sciences, Beijing, 100049 People’s Republic of China; 4 Inner Mongolia Research Center for Prataculture, the Chinese Academy of Sciences, Beijing, 100093 People’s Republic of China

**Keywords:** CBFs, Cold stress, Long non-coding RNAs, *Medicago truncatula*, *MtCIR1*

## Abstract

**Background:**

Long non-coding RNAs (lncRNAs) play important roles in the regulation of plant responses to environmental stress by acting as essential regulators of gene expression. However, whether and how lncRNAs are involved in cold acclimation-dependent freezing tolerance in plants remains largely unknown. *Medicago truncatula* is a prominent model for studies of legume genomics, and distinguished by its cold-acclimation characteristics. To determine the roles of lncRNAs in plant cold stress response, we conducted genome-wide high-throughput sequencing in the legume model plant *M. truncatula*.

**Results:**

RNA-seq data were generated from twelve samples for the four treatments, i.e., non-cold treated leaves and roots, cold-treated leaves and roots of *M. truncatula* Jemalong A17 seedlings. A total of 1204 million raw reads were generated. Of them, 1150 million filtered reads after quality control (QC) were subjected to downstream analysis. A large number of 24,368 unique lncRNAs were identified from the twelve samples. Among these lncRNAs, 983 and 1288 were responsive to cold treatment in the leaves and roots, respectively. We further found that the intronic-lncRNAs were most sensitive to the cold treatment. The cold-responsive lncRNAs were unevenly distributed across the eight chromosomes in *M. truncatula* seedlings with obvious preferences for locations. Further analyses revealed that the cold-responsive lncRNAs differed between leaves and roots. The putative target genes of the lncRNAs were predicted to mainly involve the processes of protein translation, transport, metabolism and nucleic acid transcription. Furthermore, the networks of a tandem array of *CBF/DREB1* genes that were reported to be located in a major freezing tolerance QTL region on chromosome 6 and their related lncRNAs were dissected based on their gene expression and chromosome location.

**Conclusions:**

We identified a comprehensive set of lncRNAs that were responsive to cold treatment in *M. truncatula* seedlings, and discovered tissue-specific cold-responsive lncRNAs in leaves and roots. We further dissected potential regulatory networks of *CBF* Intergenic RNA (*MtCIR1*) and *MtCBF*s that play critical roles in response and adaptation of *M. truncatula* to cold stress.

## Background

The discovery of non-coding RNAs (ncRNAs) including short (22–33 nucleotides) and long (> 200 nucleotides) ncRNAs has changed the traditional definition of a gene [1, 2]. Long non-coding RNAs (lncRNAs), which are distinguished by the lack of any obvious open reading frames (ORFs), are mainly transcribed by RNA Pol II, spliced, 5′-capped and even polyadenylated at 3′ end [2, 3]. In addition to RNA Pol II-derived lncRNAs, other classes of lncRNAs that were transcribed by two plant-specific DNA-dependent RNA polymerases, RNA Pol IV and RNA Pol V have also been reported [4]. LncRNAs can be classified into the following categories according to their genomic origins. (1) Antisense, when one or more exons of another transcript are overlapped on the opposite strand, respectively. (2) Intronic, when they are derived wholly from within an intron of a second transcript. (3) Intergenic, when they lie within the genomic intervals between two genes [2]. LncRNAs participate in the regulation of numerous biological phenomena, including those of imprinting genomic loci, shaping chromosome conformation and allosterically regulating enzymatic activity [3, 5]. LncRNAs function as key regulators of diverse mechanisms in biological processes, e.g., acting as scaffolds, decoys or signals through genomic targeting by *cis* or *trans*, and resulting in the down-regulation or overexpression of target genes [6, 7]. Studies on lncRNAs in plants have shown that they play important roles in a wide range of biological processes, especially in reproductive development and responses to environmental stresses [3, 8].

In plants, genome-wide identification of lncRNAs has been conducted in maize [9], Arabidopsis [10], Populus [11], *Medicago truncatula* [12], tomato [13] and others. The early functionally characterized plant lncRNAs are from the regulatory pathway of *FLC*, a master repressor gene involved in flowering in Arabidopsis. The lncRNAs of *COLDAIR* and *COOLAIR* negatively modulate *FLC* by different models [14–16]. A large number of lncRNAs involved in the regulation of plant responses to abiotic stresses has been characterized in recent years [17–19].

Plants grown in temperate and cold regions can enhance their tolerance to freezing by exposure to low, non-freezing temperatures for a certain period, referred to as cold acclimation [20, 21]. Numerous molecular changes during cold acclimation are responsible for cold acclimation-induced enhancement of freezing tolerance [22]. The CBF/DREB1 (C-repeat binding factor/dehydration-responsive element binding factor 1) activates the downstream CRT/DRE-containing cold-regulated (COR) genes, which in turn contributes to enhancing tolerance of plants to freezing [23–25]. *ICE1* [26], *ICE2* [27] and three closely related *CAMTAs* [28] have been identified as positive regulators of *CBF*s. The positive regulation of *RDM4* on expression of *AtCBF2* and *AtCBF3* suggests that *RDM4* is important for Pol II transcription of *CBF*s, and that it plays a critical role in tolerance of Arabidopsis to cold stress [29]. Recent studies have demonstrated that the cold-activated plasma membrane protein kinase CRPK1 phosphorylates 14–3-3 proteins, triggering its nuclear translocation to impair the stabilization of the transcription factor CBFs for a feedback of excessive cold defense response during cold stress in Arabidopsis [30]. The lncRNA *SVALKA* was identified as a negative regulator of *CBF* expression and plant freezing tolerance in Arabidopsis [31], however, how the *CBF* genes are activated by lncRNAs during cold acclimation remains to be explored.

*Medicago truncatula* is an annual forage crop [32] and has become a prominent model for legume genomics [33–35]. Given that *M. truncatula* is closely phylogenetically related to the common legume forage alfalfa (*Medicago sativa*), it is a valuable material to study molecular physiology of environmental stress in legume plants [36–38]. Alfalfa is a freezing tolerant legume species with great ability to cold acclimate, and capable of accumulating Cold-Acclimation-Specific (CAS) proteins during cold acclimation [36, 39, 40]. The *CASs* are homologous to *COR* genes that are *cis*-regulated by CBF/DREB1 factors [36, 38]. Similar to other plant species grown in temperate zones, *M. truncatula* plants have cold-acclimation traits [36, 41]. A tandem array of *CBF/DREB1* genes was located in a major freezing tolerance QTL region on chromosome 6 of *M. truncatula* [42]. To identify cold-responsive lncRNAs in *M. truncatula*, we conducted genome-wide high-throughput sequencing for four treatments (Non-cold-treated leaves, Non-cold-treated roots, Cold-treated leaves, Cold-treated roots). We identified a comprehensive set of lncRNAs that were responsive to cold treatment in *M. truncatula* seedlings. Furthermore, a possible regulatory network of *CBF* Intergenic RNA (*MtCIR1*) and *MtCBF*s in *M. truncatula* was uncovered.

## Results

### Identification of cold-responsive lncRNAs in *M. truncatula* seedlings by high-throughput sequencing

To identify cold-responsive lncRNAs*,* we conducted RNA sequencing from 12 cDNA libraries with three repeats for four treatments (Non-cold-treated leaves, NT-leaves; Non-cold-treated roots, NT-roots; Cold-treated leaves, CT-leaves; Cold-treated roots, CT-roots) of *M. truncatula* seedlings. The cDNA libraries were constructed by synthetic adaptors using mRNAs isolated from leaves and roots of three-week-old *M. truncatula* seedlings by cold treatment at 4 °C and non-cold treatment at 26 °C for 5 h, respectively. Because of the low expression levels of lncRNAs in animals and plants [2], high depth RNA sequencing was performed to generate more than 1,204,140,634 raw reads from the 12 cDNA libraries (Table 1). To assess the quality of data acquired by RNA sequencing (RNA-seq), each base in the reads was assigned a quality score (Q) by a phred-like algorithm using FastQC [43]. The results showed that the data were highly credible with Q20 higher than 95% (Supplementary Table S1). After mapping sequencing results to the *M. truncatula* A17 genome (Supplementary Table S4), a comprehensive pipeline was constructed to identify unique lncRNAs. This led to identification of more than 30,000 mRNAs and 10,000 unique lncRNAs for each one of 12 cDNA libraries (Table 1; Additional file 8: Data Set S1; Additional file 9: Data Set S2; Additional file 10: Data Set S3; Additional file 11: Data Set S4).

**Table 1:** Statistical data of the RNA-Seq results for experimental samples

Sample name	Raw reads	Clean reads	Unique mRNA	Unique lncRNA
NT_L_1	96,276,404	92,169,948	31,547	10,663
NT_L_2	97,385,326	93,513,730	34,104	13,115
NT_L_3	1.19E+ 08	1.14E+ 08	38,973	16,490
NT_R_1	92,492,642	88,455,680	37,996	13,408
NT_R_2	93,177,872	88,997,680	37,603	13,183
NT_R_3	95,007,136	90,582,052	37,976	13,435
CT_L_1	1.14E+ 08	1.09E+ 08	31,191	11,362
CT_L_2	1.1E+ 08	1.05E+ 08	30,232	10,581
CT_L_3	1.09E+ 08	1.04E+ 08	30,396	10,640
CT_R_1	89,813,904	86,248,354	37,029	13,045
CT_R_2	97,477,188	93,165,358	38,235	13,913
CT_R_3	90,282,098	86,598,790	37,414	13,542

**Fig. 1:**
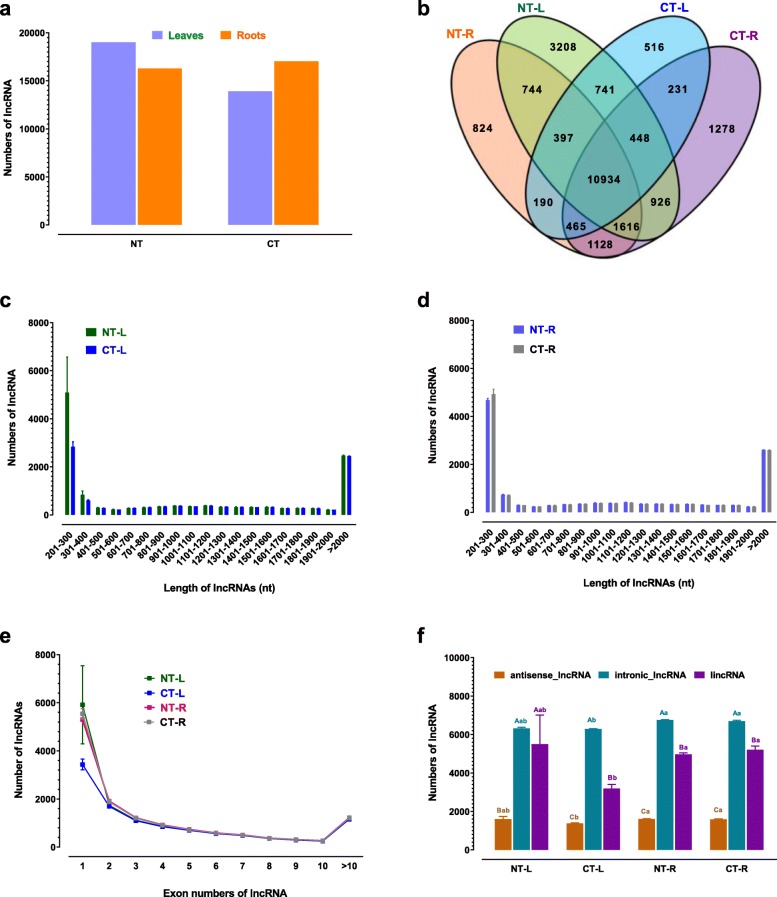
Identification of cold-responsive lncRNAs in *M. truncatula* seedlings. a: Numbers of common/specific lncRNAs identified in NT-L, CT-L, NT-R and CT-R. b: Total numbers of lncRNAs identified in leaves (NT, CT) and roots (NT, CT). Values shown in (**a**) and (**b**) were obtained from pooling data of three independent RNAseq experiments. Length distribution of lncRNAs in leaves (**c**) and roots (d). e: Numbers of lncRNAs containing different numbers of exon in NT-L, CT-L, NT-R and CT-R. f: Numbers of three type lncRNAs identified in NT-L, CT-L, NT-R and CT-R. Capital letters indicate a significant different at *p* < 0.05 according to *t*-test between three type lncRNAs in same treatment samples and small letters indicate a significant different at *p* < 0.05 according to *t*-test between NT-L, CT-L, NT-R and CT-R of same type lncRNAs. Values shown in (**c**), (**d**), (**e**) and (**f**) are means ± SE with three independent RNAseq experiments. Antisense-lncRNA: lncRNA overlapping with reference on the opposite strand; intronic-lncRNA: lncRNA falling entirely within a reference intron; incRNA: intergenic lncRNA; NT: non-cold treated; CT: cold treated; L: leaves; R: roots

To identify total numbers of lncRNAs in the four treatment samples, we merged data of three biological repeats (Correlation coefficient was shown in Supplementary Table S2 and S3) for each treatment regardless of repeatability, and 19,014, 16,298, 13,922 and 17,026 unique lncRNAs were identified in NT-leaves, NT-roots, CT-leaves and CT-roots, respectively (Fig. 1a). The numbers of identified lncRNAs in leaves were greater than those in roots of NT (19,014/16,298) scenarios, but they were less after cold treatment (13,922 vs 17,026) (Fig. 1a). The numbers of identified lncRNAs in CT-leaves were reduced by 5092, which resulted from 6494 disappearance and 1402 appearance compared with NT-leaves. In contrast, the numbers of identified lncRNAs were increased by 728 in CT-roots, which resulted from 2155 disappearance and 2883 appearance compared with NT-roots (Fig. 1a).

We identified 24,368 unique lncRNAs by pooling data from four treatments with 12 sample libraries (Fig. 1b). Among these lncRNAs, we simultaneously identified 10,934 (44.9%) unique lncRNAs in the four treatments, and 4465 (18.3%) and 3230 (13.3%) lncRNAs were specific to leaves and roots, respectively (Fig. 1b). In addition, 3208 (13.2%) and 516 (2.1%) lncRNAs were specifically identified in non-cold-treated and cold-treated leaves, and 824 (3.4%) and 1278 (5.2%) in non-cold treated and cold treated roots, respectively (Fig. 1b).

In terms of the length, the majority of lncRNAs was relatively short, such that the percentage of lncRNAs shorter than 2000 nt accounted for 81.1, 77.4, 80.5 and 80.8% in NT-leaves, NT-roots, CT-leaves and CT-roots, respectively (Fig. 1c and d). Specifically, we found that lncRNAs shorter than 400 nt were dominant ones. For example, lncRNAs shorter than 400 nt were 42.6, 31.6, 40.8 and 41.8% of total lncRNAs in NT-leaves, NT-roots, CT-leaves and CT-roots, respectively (Fig. 1c and d).

We also analyzed exon numbers in the lncRNAs. As shown in Fig. 1e, the numbers of lncRNAs dramatically reduced with increasing exon numbers, and the numbers of lncRNAs containing only one exon were the most, and accounted for about 42.4, 39.8, 31.5 and 41.0% in NT-leaves, NT-roots, CT-leaves and CT-roots, respectively (Fig. 1e).

In the present study, we classified the lncRNAs into three categories, i.e., antisense-lncRNA (lncRNA overlapping with reference on the opposite strand), intronic-lncRNA (lncRNA falling entirely within a reference intron) and lincRNA (intergenic lncRNA) according to their genomic origins (Fig. 1f). Among the three types of lncRNAs, the most and least numbers belonged to intronic-lncRNA and antisense-lncRNA ones in both leaves and roots (Fig. 1f).

Analyses of lncRNAs distribution on chromosome revealed that the identified lncRNAs were unevenly distributed across the eight chromosomes in *M. truncatula* seedlings with obvious preferences for locations (Additional file 2: Fig. S1; Additional file 3: Fig. S2).

### Characterization of cold-responsive lncRNAs in *M. truncatula* seedlings

We characterized the cold-responsive lncRNAs according to the following five aspects: change fold (FC(log_2_)), exon number, length of lncRNAs, distribution on chromosome, and type of lncRNAs in leaves and roots of *M. truncatula*. The lncRNAs displaying different expression were selected based on change fold≥2 of TPM (Transcripts Per Million) and *p*-value< 0.05 for three biological repeats between cold and non-cold treated leaves and roots (Additional file 8: Data Set S1).

**Fig. 2:**
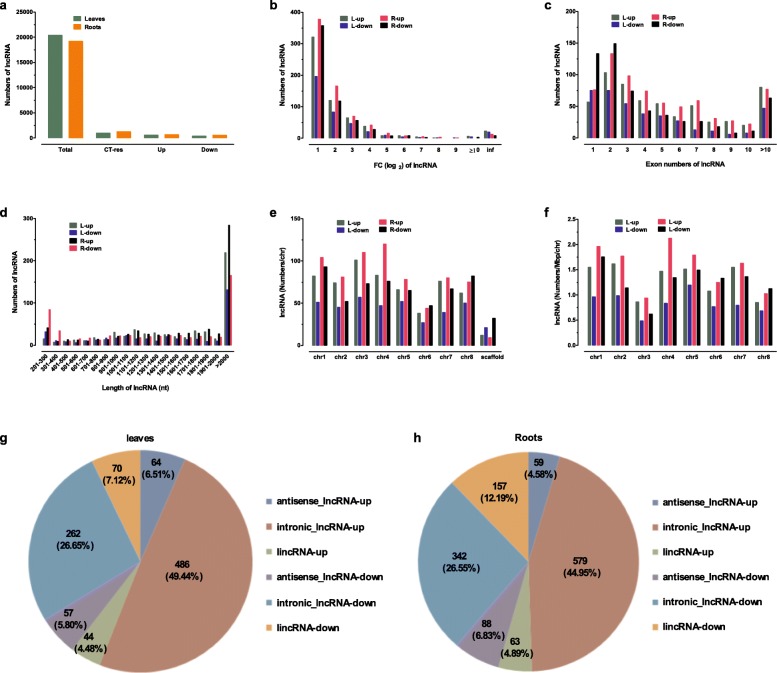
Characteristics of cold-responsive lncRNAs in *M. truncatula* seedlings. **a**: Numbers of cold-responsive lncRNAs in leaves and roots of *M. truncatula* seedlings. **b**: Numbers of lncRNAs with different FC(log2) which were regulated by cold treatment in leaves and roots of *M. truncatula* seedlings. Data on X axis indicated varying (FC(log2)) values ranging from ≥1 to ≥10. **c**: Numbers of lncRNAs containing different exon numbers in response to cold treatment in leaves and roots of *M. truncatula* seedlings. d: Numbers of lncRNAs with different length responded to cold treatment in leaves and roots of *M. truncatula* seedlings. **e**: Numbers of lncRNAs distributed on chromosomes in response to cold treatment in leaves and roots of *M. truncatula* seedlings. **f**: Densities of lncRNAs on 8 chromosomes responded to cold treatment in leaves and roots of *M. truncatula* seedlings. CT-res: cold treatment responsive-lncRNAs; up- or down-regulated: expression of lncRNAs induced or reduced by cold treatment; L: leaves; R: roots. Three types of lncRNAs (antisense-lncRNA, intronic-lncRNA and lincRNA) regulated by cold treatment in leaves (**g**) and roots (**h**) of *M. truncatula* seedlings. Antisense-lncRNA: lncRNA overlapping with reference on the opposite strand; intronic-lncRNA: lncRNA falling entirely within a reference intron; lincRNA: intergenic lncRNA

Among identified lncRNAs, 983 and 1288 were responsive to cold treatment in the leaves and roots, respectively (Fig. 2a). The numbers of cold-responsive lncRNAs were greater in roots than those in leaves as well as those of up- or down-regulated lncRNAs by cold treatment (Fig. 2a). The numbers of lncRNAs that were up-regulated were greater than those of down-regulated lncRNAs in both leaves and roots (Fig. 2b). Moreover, only a few lncRNAs were specifically found to be up-regulated by cold treatment in cold treated leaves and roots (Additional file 8: Data Set S1). As shown in Fig. 2b, the numbers of lncRNAs with expression levels of up- or down-regulated were reduced with increasing (FC(log_2_)) in both leaves and roots. The highest numbers of up- or down-regulated lncRNAs were those whose expression changes were FC(log_2_) < 4. For example, they were 91.6 and 89.2% for up-regulated and down-regulated in leaves, while they were 93.6 and 95.2% for up-regulated and down-regulated in roots, respectively (Fig. 2b).

The numbers of lncRNAs showing up- or down-regulation were also reduced with increasing exon numbers except lncRNAs containing two exons in either leaves or roots (Fig. 2c). Different from the results that the numbers of identified lncRNAs containing only one exon were most in the four treatments, the most numbers of cold-responsive lncRNAs were those containing two exons (Fig. 2c). Although the majority (about 40%) of lncRNAs identified in *M. truncatula* seedlings was shorter than 400 nt (Fig. 2c and d), lncRNAs shorter than 2000 nt showed comparable responses to cold treatment regardless of their length (Fig. 2d).

The numbers of up-regulated lncRNAs were more than those of down-regulated lncRNAs in leaves across the eight chromosomes in response to cold treatment (Fig. 2e and f). In contrast, the cold-responsive lncRNAs exhibited different patterns in roots. For example, the numbers of up-regulated lncRNAs in roots were less than those of down-regulated lncRNAs on chr 6 and chr 8, while the numbers of up-regulated lncRNAs were more than those of down-regulated lncRNAs on the remaining chromosomes in response to cold treatment (Fig. 2e and f). Further analyses indicated that cold treatment-induced increases in number and density of identified lncRNAs differed among 8 chromosomes in leaves and roots (Fig. 2e and f). The largest number was found on chr3 (17.0%), while the fewest one was on chr6 (6.4%) in leaves (Fig. 2e). The most number of lncRNAs up-regulated by cold treatment was on chr4 (20.2%), while the fewest one was on chr6 (21.1%) in roots (Fig. 2e). Although the chromosomes with the highest density of up- and down-regulated lncRNAs in leaves differed from those in roots, the chromosomes with the lowest density of up- and down- regulated lncRNAs were on chr3 in both leaves and roots (Fig. 2e and f). These results suggest that each chromosome has distinct patterns in response to cold treatment in term of expression of lncRANs.

The most numbers of cold-responsive lncRNAs belonged to intronic-lncRNAs both in leaves and roots, but the least ones were linc-lncRNAs in leaves and antisense-lncRNAs in roots (Fig. 2g and h). Moreover, different types of lncRNAs differed in their responses to cold treatment, such that the expression of 49.4 and 45.0% of intronic-lncRNAs in leaves and roots was up-regulated by cold treatment, but it was only 6.5 and 4.9% for antisense-lncRNAs, and 4.5 and 4.9% for linc-lncRNAs, respectively (Fig. 2g and h). Similar to the up-regulated lncRNAs, the numbers of down-regulated intronic-lncRNAs were also most, but the least ones were antisense-lncRNAs in both of leaves and roots (Fig. 2g and h). These results may suggest that intronic-lncRNAs are most sensitive lncRNAs to cold treatment.

### Functional characterization of cold-responsive lncRNAs in *M. truncatula* seedlings

**Fig. 3:**
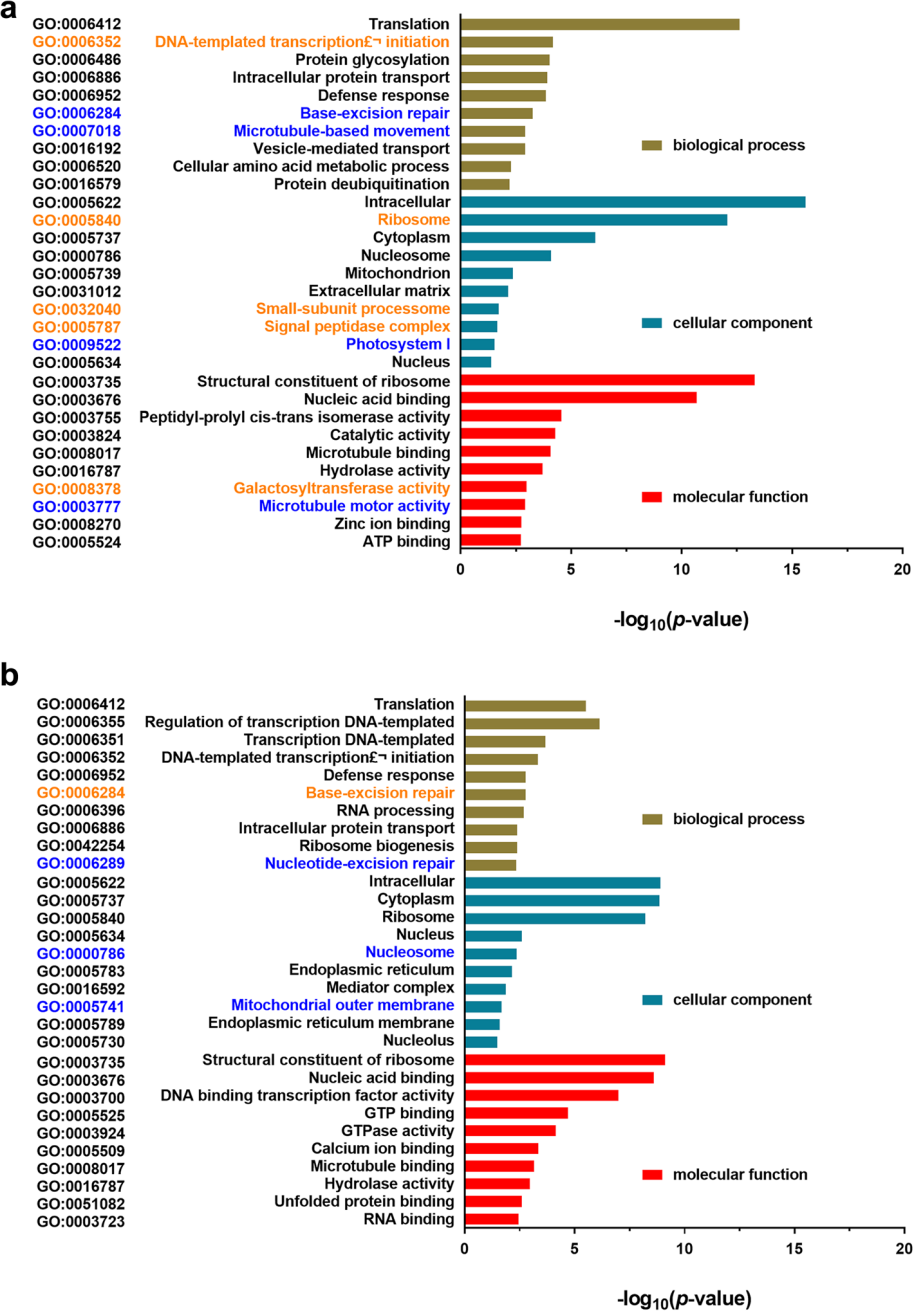
Gene Ontology (GO) analysis. GO terms of genes that were co-expressed and co-localized with the cold-responsive lncRNAs based on transcriptional expression and genomic location in leaves (**a**) and roots (**b**) of *M. truncatula* seedlings. The reliability was calculated by –log10 (*p* < 0.05). Black color words in Y-axis indicated the genes of these GO terms were up- or down-regulated by cold treatment. Orange color words in Y-axis indicated the genes of these GO terms were up-regulated by cold treatment. Blue color words in Y-axis indicated the genes of these GO terms were down-regulated by cold treatment. More information is detailed in Additional file 1: Table S5 and S6

To uncover potential functions of the cold-responsive lncRNAs, we analyzed Gene Ontology (GO) terms of genes that were co-expressed and co-localized with the cold-responsive lncRNAs based on the transcriptional expression of three biological repeats between cold and non-cold treated leaves and roots and genomic location in 100 kb. The top 10 terms of biological processes, cellular components and molecular functions were analyzed according to significant enrichments (corrected *P* < 0.05) in leaves and roots (Fig. 3; Additional file 1: Table S5 and S6).

Among the top 10 terms of biological processes, expression of all genes associated with DNA-templated transcription (initiation) (GO:0006352) in leaves and those associated with base-excision repair (GO:0006284) in roots was induced by cold treatment. Expression of all genes involved in base-excision repair (GO:0006284) and vesicle-mediated transport (GO:0007018) in leaves and genes in nucleotide-excision repair (GO:0006289) in roots was reduced by cold treatment. Genes in the majority of GO groups were up- or down- regulated by cold treatment (Fig. 3). For the top 10 terms of cellular components in leaves, cold treatment up-regulated expression of all genes in the three GO groups of leaves, i.e., ribosome, small-subunit processome and signal peptidase complex (GO:0005840, GO:0032030, GO:0005787). In contrast, cold treatment led to down-regulation of expression of all genes in nucleosome (GO:0000786) and mitochondrial outer membrane (GO:0005741) in roots. Genes in other GO groups of cellular component were up- or down regulated by cold treatment in either leaves or roots (Fig. 3). The molecular function of genes co-expressed and co-localized with cold-responsive lncRNAs in leaves and roots were extremely complicated. Expression of genes in galactosyl transferase activity group (GO:0008378) was induced, and expression of genes in microtubule motor activity group (GO:0003777) was reduced by cold treatment (Fig. 3). Proteins encoded by these mRNAs had different functions in leaves and roots, suggesting organ-specific responses to cold treatment. These results highlight the diversity of lncRNAs genomic location and complexity of regulation function acting on their potential target genes.

**Fig. 4:**
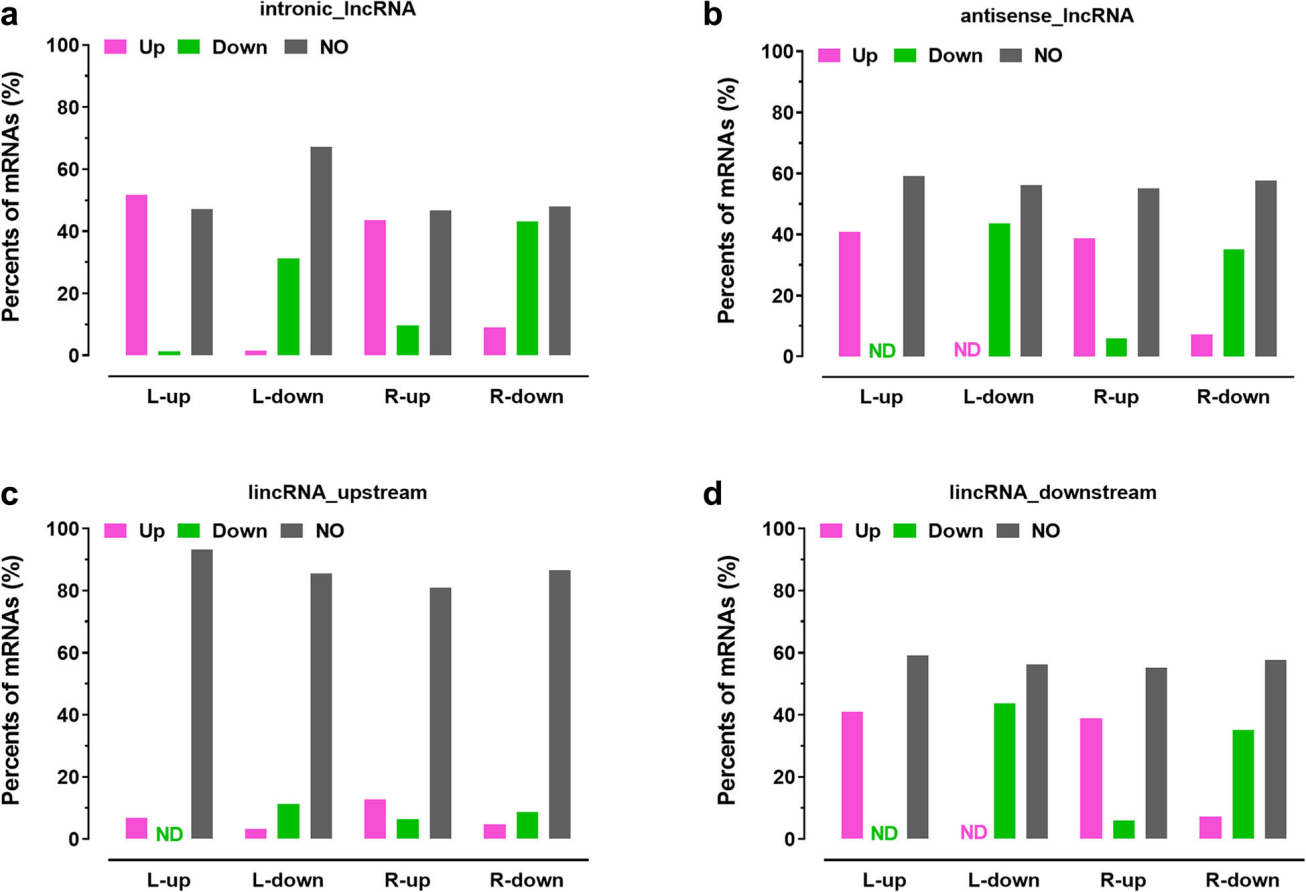
Correlation analyses of lncRNAs and their potential target genes in *M. truncatula*. Expression models of intronic-lncRNAs (**a**), antisense-lncRNAs (**b**) and their potential target genes in leaves and roots of *M. truncatula* seedlings responsive to cold treatment. Expression models of lincRNAs and their potential upstream target genes (**c**), downstream genes (**d**) in leaves and roots of *M. truncatula* seedlings responding to cold treatment. The expressions were analyzed by RNA-seq (TPM). Antisense-lncRNA: lncRNA overlapping with reference on the opposite strand; intronic-lncRNA: lncRNA falling entirely within a reference intron; lincRNA: intergenic lncRNA; L: leaves; R: roots; up: expression were induced by cold treatment; down: expression were reduced by cold treatment; ND: no detected

To further characterize the expression relationship between lncRNAs and their potential target protein-coding genes, correlations between expression patterns of lncRNAs and their potential target genes that were responsive to cold treatment were analyzed (Fig. 4). As shown in Fig. 4, more than 30% of potential target genes displayed similar expression patterns with intronic-lncRNAs and antisense-lncRNAs (Fig. 4a and b). For example, about 51.7% potential target genes of cold-induced up-expression of intronic-lncRNAs in leaves (L-up) were detected, while 43.6% potential target genes of cold-induced expression of intronic-lncRNAs in roots (R-up) were found. Similar to cold-induced expression of lncRNAs, 31.3% potential target genes of L-down (i.e., expression was reduced by cold treatment in leaves) intronic-lncRNAs were reduced by cold treatment, while it was 43.1% for potential target genes of R-down (i.e., expression was reduced by cold treatment in roots) intronic-lncRNAs (Fig. 4a). Similar scenarios were found for those potential target genes of antisense-lncRNAs (Fig. 4b). In addition, the expression patterns of potential target genes related with lincRNAs were more complex (Fig. 4c and d). The expression patterns of upstream potential target genes exhibited little similarity to their related lincRNAs (Fig. 4c). On the contrary, the expression of downstream potential target genes was positively related with expression of adjacent lincRNAs (Fig. 4d). For example, about 40.8% downstream potential target genes of L-up lincRNAs were induced by cold treatment, while 38.8% downstream potential target genes of R-up lincRNAs were induced by the same cold treatment. Similar to cold-induced expression of lncRNAs, 43.8% downstream potential target genes of down-regulated lincRNAs in leaves (L-down) were reduced by cold treatment, while 35.1% downstream potential target genes of down-regulated lincRNAs in roots (R-down) (Fig. 4d).

### Analyses of lncRNA-*MtCBF*s networks

**Fig. 5:**
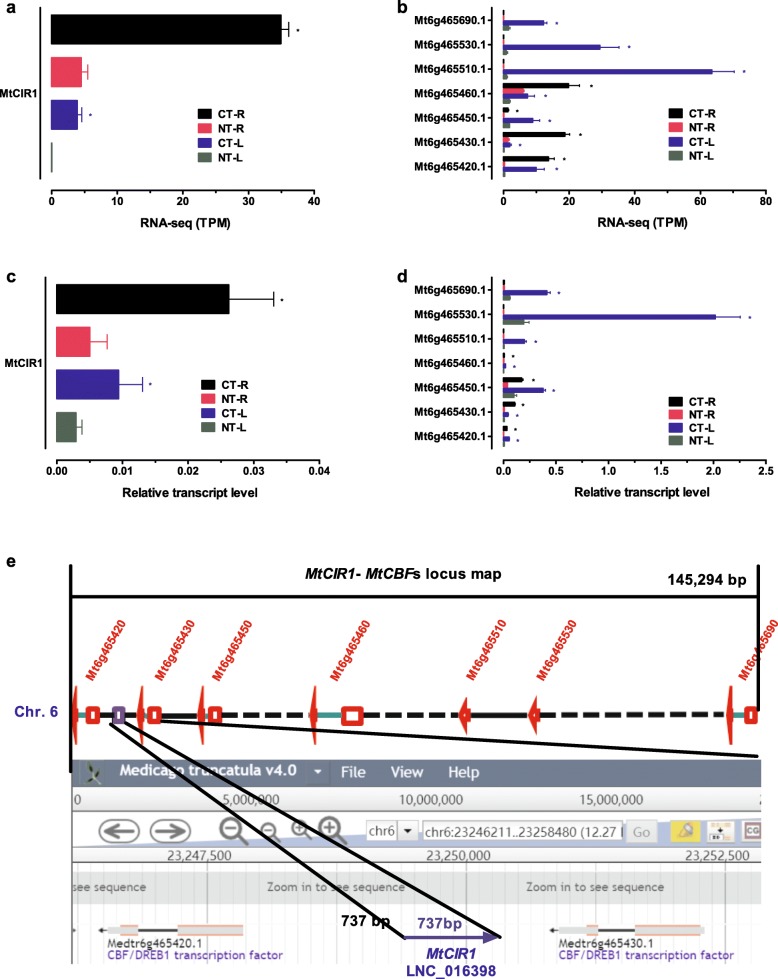
Relationship between lncRNA *MtCIR1* and their potential target *MtCBF* genes in *M. truncatula* plants. Transcriptional responses of *MtCIR1* (**a**) and *MtCBF* genes (**b**) in *M. truncatula* plants to cold treatment at 4 °C for 5 h analyzed by RNA-seq (TPM). Transcription responses of *MtCIR1* (**c**) and *MtCBF* genes (**d**) in *M. truncatula* plants to cold treatment at 4 °C for 5 h verified by real-time quantitative Q-PCR. NT: non-cold treated; CT: cold treated; L: leaves; R: roots. Values are means ± SE (*n* = 3) and the asterisks indicate significant differences at *p* < 0.05 between cold-treated sample (CT) and non-cold-treated control (NT) according to *t*-test. e: Genome map of the *MtCBF* gene clusters and *MtCIR1*. *MtCIR1* and *MtCBF* gene clusters are located in a 145.3 kb on chromosome 6 of *M. truncatula* Jemalong A17. Red rectangle indicates exon. Red arrow indicates exon and direction of gene from 5′ to 3′. Green line indicates intron. Blue rectangle indicates lncRNA. Black line indicates intergenic region. Black numbers indicate length (bp) of intergenic region

C-repeat/DRE binding proteins (*CBF*s) have been identified to be pivotal transcription regulatory factors of cold-responsive (*COR*) genes during cold treatment in many plant species [22]. A tandem array of *CBF/DREB1* genes has been reported to be located in a major freezing tolerance QTL region on chromosome 6 in *M. truncatula* [42]. To determine the roles of cold-responsive lncRNAs in regulating *MtCBF*s, we monitored the expression patterns of lncRNAs and *MtCBF*s by taking into account of their locations on the chromosomes. One reverse-direction transcription lncRNA (LNC_016398-*MtCIR1*, *M.truncatulaCBF*s Intergenic RNA) (Fig. 5a and c) and 7 *CBF* genes (*Mt6g465420*, *Mt6g465430*, *Mt6g465450*, *Mt6g465460*, *Mt6g465510*, *Mt6g465530* and *Mt6g465690*) (Fig. 5b and d) were induced by cold treatment by RNA-seq (Fig. 5a and b), and verified by real-time quantitative Q-PCR (Fig. 5c and d). Analyses of genomic location of the *MtCIR1* and seven *CBF* genes indicated that they were neighborly distributed on chromosome 6 (Fig. 5e). *MtCIR1* (733 bp) was located in the intergenic region of *Mt6g465420* and *Mt6g465430*, and it did not show any overlap with these genes (Fig. 5e).

**Fig. 6:**
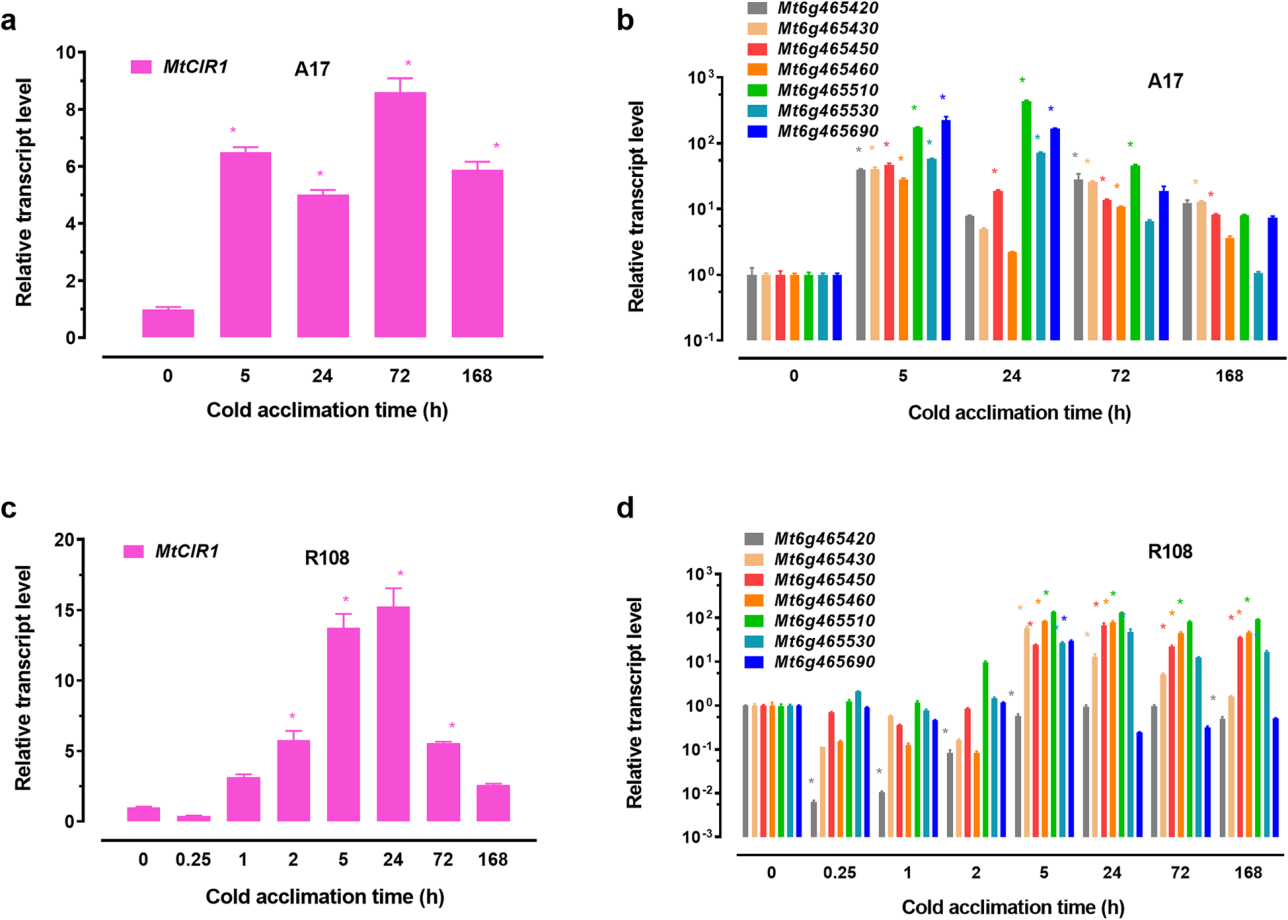
Responses of lncRNA *MtCIR1* and their potential target *MtCBF* genes to cold treatment. **a**: Transcriptional responses of *MtCIR1* in leaves of *M. truncatula* seedlings subjected to cold treatment. **b**: Transcriptional responses of *MtCBF* genes in leaves of *M. truncatula* seedlings subjected to cold treatment. Leaf RNA was extracted from 3-week-old seedlings cold treated at 4 °C for varying time periods and then the relative expression was detected using real-time quantitative RT-PCR. Values are means ± SE (n = 3) and the asterisks indicate significant differences at *p* < 0.05 according to *t*-test relative to room temperature control (0 h)

lncRNA *MtCIR1* was not only closely neighbored by the seven *MtCBF*s on the chromosome 6 (Fig. 5e), its expression level was also comparable to that cold-induced *MtCBF*s (Fig. 5a-d)*.* To confirm the results, we tested transcript responses of *MtCIR1* and their potential target *MtCBF* genes to cold treatment by quantitative real-time PCR in leaves of *M. truncatula* (Fig. 6). The expression of *MtCIR1* was up-regulated by cold treatment (Fig. 6a). For instance, after cold treatment for 5 h, transcripts of *MtCIR1* was increased by 7 fold (Fig. 6a). Transcripts of the seven *MtCBF*s that were located on chr6 and neighbored by the *MtCIR1* were transiently increased in leaves (Fig. 6b). An important question is whether the cold treatment-induced expression of *MtCIR1* preceded the expression of *MtCBF*s. To answer this question, we monitored the expression of *MtCIR1* and *MtCBF*s during early stage (0 to 2 h) of cold treatment in leaves. As shown in Fig. 6a, the transcript levels of *MtCIR1* were induced markedly within 2 h of cold treatment. The cold-induced increase in *MtCIR1* was followed at 5 h by accumulation of transcripts for *MtCBF*s (Fig. 6b). In contrast to other four *MtCBF*s, which peaked at 5 h and then fall during cold treatment, the expression of three *MtCBF*s, *Mt6g465510*, *Mt6g465530* and *Mt6g465690* also peaked at 5 h and remained at the high level during cold treatment up to 24 h (Fig. 6b). The results that up-expression of *MtCIR1* was followed by the induction of *MtCBF* transcripts during cold treatment may suggest the existence of crosstalk between the lncRNA *MtCIR1* and *MtCBF* genes.

## Discussion

Studies on lncRNAs in plants have shown that they play important roles in a wide range of biological processes, especially in regulating plant responses to biotic and abiotic stress, such as drought stress in maize [9] and *Populus trichocarpa* [11], salt and drought stress in *Medicago truncatula* [12], *P. infestans*-resistant in tomatoes [13]. However, we know little about whether involvement of lncRNAs in cold acclimation-dependent freezing tolerance in legume model plant *Medicago truncatula*. In the present study, we identified 24,368 unique lncRNAs that is similar to numbers obtained in *M. truncatula* induced by salt and drought stress lncRNAs [12]. Among 24,368 lncRNAs, 983 and 1288 were responsive to cold treatment in the leaves and roots. The cold-responsive lncRNAs found in our study are more than those identified in cassava, and this may result from the different sequencing methods and plant species, such as different genome sizes and sensitivities to low temperature stress [44].

Our results showed that total numbers of identified unique lncRNAs and mRNAs in leaves were greater than those in roots, but the proportion of cold-responsive lncRNAs and mRNAs was higher in roots than that in leaves (Figs. 1 and 2; Additional file 4: Fig. S3; Additional file 5: Fig. S4). These results may suggest that different organs have specific responses to cold treatment, such that lncRNAs and mRNAs in roots may be more sensitive to cold treatment. The higher proportion of cold-responsive lncRNAs and mRNAs in roots relative to those in leaves may imply the greater importance of roots in response to cold treatment. However, few studies have focused on physiological and molecular responses of roots to cold stress so far [45, 46]. It has been suggested that the capacity for cold acclimation and frost resistance in four annual legumes were related to a higher root to shoot ratio and higher concentrations of solutes in roots [47]. Therefore, root-mediated physiological processes may play important roles in the regulation of cold treatment-dependent freezing tolerance in plants.

Our results that the majority of GO terms was involved in the regulation of varying biological processes are consistent with the reports suggesting existence of complicated mechanisms in plant cold stress response [21, 48]. GO terms involved in defense response may directly participate in the protection of cells under stress conditions. The GO term of 0006355 associated with the regulation of transcription has been reported to play a role in response to freezing stress in *M. sativa* [49]. It has been reported that the gene coding for Cu/Zn superoxide dismutase, which is cleaved by miR398 in plants, was involved in the regulation of cold-stress responses by acting as a scavenger of reactive oxygen species (ROS) [50, 51]. These results may imply that correlations between lncRNAs and microRNAs may exist in the modulation of responses to cold stress. An interesting finding is that genes included in base-excision repair (GO: 0006284) were down-regulated in leaves, but up-regulated in roots by cold treatment (Fig. 3). DNA damaging agents can be a great threat to genomes. To protect DNA from the damage, a number of DNA repairing strategies have been developed. The base- excision-repair pathway is a highly conserved mechanism in the organisms, and has been suggested to account for removal and repair of mutagenic oxidative DNA lesions [52]. Although there are extensive studies on the roles of base excision repair system in stress and plant hormone signaling [53, 54], little is known about their effects on plant cold stress response. The GO terms of base-excision repair identified in this research indicate that the base-excision repair may play important roles in repairing DNA during cold treatment.

As key transcriptional activators, the gene family of dehydration and cold response (*CBF/DREB1*) activates the downstream cold-regulated (*COR*) genes, which in turn contributes to enhancement of tolerance to freezing stress [22, 24, 55]. A major freezing tolerance QTL (Mt-FTQTL6) accounting for 40% of the phenotypic variation among 15 *M. truncatula* accessions has been mapped to a region of *M. truncatula* chromosome 6 [56]. A tandem array of *CBF/DREB1* genes was located in the major freezing tolerance QTL (Mt-FTQTL6) region on the chromosome 6 in *M. truncatula* [42]. However, as an early event of a low temperature-stimulated signaling cascade, how the expression of *CBF*s is induced by cold treatment remains largely unclear. Numerous reports have demonstrated that lncRNAs participate in the regulation of various biological processes by interacting with DNA and RNA molecules, and transcription factors, leading to alterations of target genes [5]. Furthermore, the expression patterns of lncRNAs are often correlated with those of mRNA in both *cis* and *trans* manners, suggesting that certain lncRNAs may be co-regulated in the expression networks [57]. In the present study, we found the involvement of lncRNAs and *MtCBF*s genes (GO: 0005634) in cold response by RNA-sequencing and gene expression analyses. We demonstrated that expression of six *MtCBF*s was related to the expression of one lncRNA *MtCIR1*. Specifically, we found that *MtCIR1* was an intergenic noncoding RNA located in a close proximity to *MtCBF* genes, and that the expression of the *MtCIR1* and *MtCBF* genes was induced by cold treatment. This observation is in line with a report demonstrating that about half of the intergenic noncoding RNAs is transcribed close to protein-coding genes [57]. Therefore, these data may provide important clues for further dissection about the transcription regulation of intergenic noncoding RNAs on neighboring genes during cold treatment.

The observations that transcript levels of lncRNA *MtCIR1* increased within 2 h of exposure to low temperature, followed by accumulation of *MtCBF*s at 5 h may suggest a regulatory network between *MtCBF*s and *MtCIR1*. However, these results differ from previous studies that the accumulation of *AtCBF1–3* transcripts was detected within 15 min of plants upon exposure to low temperature, followed by accumulation of *COR* gene transcripts at 2 h [58]. Despite the high similarities in sequence and close evolution relationships between the six *MtCBF*s identified in this research with *AtCBF1–3*, the difference in cold-response may imply that the sensitivity of *CBF*s transcript to cold treatment is specific to plant species. For example, Arabidopsis plants are more sensitive to cold stress than *M. truncatula* plants (Additional file 6: Fig. S5; Additional file 7: Fig. S6). Recent studies found an lncRNA *SVALKA* in a cold-sensitive region of the Arabidopsis genome. Mutations in *SVALKA* affect CBF1 expression and freezing tolerance [31]. Whether lncRNA *MtCIR1* in *M. truncatula* has similar function in the regulation of plant freezing tolerance with *SVALKA* in Arabidopsis warrants further studies using genetics and physiology methods.

Many studies have found that *ICE1*, *ICE2*, the three closely related *CAMTA*s, and *RDM4*, are positive regulators of *CBF*s, and that the phosphorylated 14–3-3 proteins destabilize CBF proteins [26–30, 59]. However, whether *MtCIR1* is correlated with these proteins in the regulation of *MtCBF*s and freezing tolerance remains to be elucidated. Shu et al. [49] identified a number of freezing- and *Medicago*-specific miRNAs involved in the regulation of freezing tolerance in *M. sativa*. One important mechanism of lncRNAs at the post-transcriptional is to function as mediators, including functioning as the precursors of small RNAs, and acting with miRNAs to regulate mRNA turnover [60]. Whether the cold responsive *MtCBF*s and lncRNA *MtCIR1* found in the present study is also related with these microRNA remains to be uncovered in the future study.

The expression levels of sequencing and qPCR were inconsistent in our research, especially at expression levels of lncRNAs (Fig. 5a-d). There are mainly three factors that may account for these results. Firstly, a certain degree of inconsistency between the results obtained by the two methods may be expected, especially for the low expression of RNAs because the sequencing and qPCR are two different detection methods. Secondly, qPCR was used to verify the sequencing results, which verified the difference trend, i.e. up-regulated or down-regulated, rather than the difference multiple. Lastly, duplication during sequencing might partly interfere quantitative results of RNA transcripts [61, 62].

## Conclusions

In the present study, we identified a large number of cold-responsive lncRNAs in both leaves and roots of legume model plant *M. truncatula* seedlings. We further demonstrated that the cold-responsive lncRNAs were tissue-specific, i.e., the numbers of identified lncRNAs in leaves were greater than those in roots of non-cold stress scenarios, but they were less after cold treatment. We found that the three types of intronic-lncRNAs differed in their responses to cold stress, with the intronic-lncRNAs being the most sensitive to cold stress. Another interesting finding is that cold-responsive lncRNAs were unevenly distributed across the eight chromosomes in the genome of *M. truncatula* with obvious preferences for locations. Furthermore, we dissected a regulatory network of lncRNA-*MtCBF*s that may play a critical role in response and adaptation of plants to cold stress by integrating lncRNA *MtCIR1* with their potential target *MtCBF* genes.

## Methods

### Plant materials and growth conditions

Seeds of *M. truncatula,* Jemalong A17 used in this work were kindly provided by Dr. Carroll Vance, USDA-ARS, Plant Sceience Research, St. Paul, MN, USA. The Jemalong A17 is the model plant of legume plants, whose genome has been sequenced [35, 63]. Seeds of *Medicago truncatula* were treated with concentrated sulfuric acid for 4 min, and then thoroughly rinsed with water. After chilled at 4 °C for 2 d, seeds were sown on 0.8% agar to germinate at 25 °C till the radicals being about 2 cm. The seedlings were planted in the plastic buckets (three seedlings in one bucket) filled with aerated nutrient solution. The composition of full-strength nutrient solution is: 2.5 mM KNO_3_, 0.5 mM KH_2_PO_4_, 0.25 mM CaCl_2_, 1 mM MgSO_4_, 100 μM Fe-Na-EDTA, 30 μM H_3_BO_3_, 5 μM MnSO_4_, 1 μM ZnSO_4_, 1 μM CuSO_4_ and 0.7 μM Na_2_MoO_4_ with pH of 6.0. Plants were grown in green house under 26 °C day/22 °C night, and 14-h photoperiod, 120 μmolm^− 2^ s^− 1^ conditions.

### Cold treatment and sample collection

Nine three-week-old Jemalong A17 seedlings grown in three plastic buckets were cold treated at 4 °C, 60 μmolm^− 2^ s^− 1^ for 5 h (CT). In the meanwhile, nine three-week-old Jemalong A17 seedlings grown in another three plastic buckets transferred to 26 °C, 60 μmolm^− 2^ s^− 1^ for 5 h were used as control (NT). Nine leaflets (randomization approach) for leaf-samples and three entire roots for root-samples from three plants grown in one plastic bucket were collected for one biological replicate, respectively. Three biological replicates were maintained for sample collection by a temperature treatment was repeated three separate times in a single growth chamber under same condition. Finally, twelve samples for four treatments of non-cold treated leaves and roots (NT-L, NT-R), cold-treated leaves and roots (CT-L, CT-R) were collected for RNA sequencing. In the meanwhile, twelve sub-samples were collected for qRT-PCR test. After harvest, samples were frozen in liquid nitrogen, and stored at − 80 °C for RNA extraction.

### RNA extraction and cDNA library construction

Total RNAs were extracted from different samples using the Trizol (Invitrogen) according to the manufacturer’s protocols. After DNA digestion with RNase-free DNase I (Promega). RNA degradation and contamination were evaluated on 1% agarose gels. The purity of RNA was checked by the NanoPhotometer® spectrophotometer (IMPLEN, CA, USA). RNA concentration was determined by Qubit® RNA Assay Kit in Qubit® 2.0 Flurometer (Life Technologies, CA, USA). RNA integrity was checked by the RNA Nano 6000 Assay Kit (Bioanalyzer 2100, Agilent Technologies, CA, USA).

For each sample, an amount of 3 μg RNA was used for the preparation of RNA samples. Ribosomal RNA was first removed by Epicentre Ribo-zero™ rRNA Removal Kit (Epicentre, USA), and rRNA free residue was cleaned by ethanol precipitation. Thereafter, the sequencing libraries were generated using the rRNA-depleted RNA by NEBNext® Ultra™ Directional RNA Library Prep Kit for Illumina® (NEB, USA) following the protocols described by the manufacturer. Products were purified (AMPure XP system) and library quality was determined with the Agilent Bioanalyzer 2100 system.

### Sequencing and data analysis

The libraries were sequenced on an Illumina Hiseq 4000 platform and 150 bp paired-end reads were generated. The raw reads from the 12 samples were used for quality filtering. Clean reads were obtained by removal of reads containing adapter, ploy-N and low quality reads from raw data. At the same time, Q20, Q30 and GC contents of the clean data were determined [43]. The low quality reads (Phred score < 20; read length < 50 bases) and reads with adapter contamination were removed to generate a set of high quality reads termed as clean data thereafter. All the downstream analyses were based on the clean data with high quality.

### Reads mapping and transcriptome assembling

The clean reads mapped to the *M. truncatula* genome. Index of the *Medicago* Genome Sequences V4.0 (http://www.medicagohapmap.org/tools/blastform) was built using bowtie2 v2.2.8 and paired-end clean reads were aligned to the *M. truncatula* genome using HISAT2 v2.0.4 [64]. To construct transcriptome, the mapped reads were assembled de novo using Cufflinks [65]. All transcripts were required to be > 200 bp in length.

The mapped reads of each sample were assembled by StringTie (v1.3.1) [66] in a reference-based approach. StringTie uses a novel network flow algorithm as well as an optional de novo assembly step to assemble and quantitate full-length transcripts representing multiple splice variants for each gene locus.

### Coding potential analysis and identification of lncRNAs

Predication of transcripts with coding potential was made using the following tools, including CNCI (Coding-Non-Coding-Index) (v2)) [67], CPC (Coding Potential Calculator) (0.9-r2) [68], Pfam Scan (v1.3) [69] and PhyloCSF (phylogenetic codon substitution frequency) (v20121028) [70]. The left unknown transcripts without coding potential were taken as candidate set of lncRNAs.

### Target gene prediction

We first identified coding genes that were located 100 k upstream and downstream of lncRNA, and then analyzed their functions. The genes from different samples were clustered with WGCNA (Weighted Gene Co-expression Network Analysis) [71] to search for common expression modules, and their function was analyzed by the functional enrichment analysis.

### Quantification of gene expression level and analysis of differential expression

Kallisto-sleuth pipelines was used to calculate TPMs of both lncRNAs and coding genes in each sample [72]. TPM means transcript per kilobase of exon per million fragments mapped, calculated based on the length of the transcript and reads count mapped to this transcript. The differentially expressed genes (DEGs) with log2 fold change ≥2 (induced) and/ or ≤ − 2 (reduced) and a *P* value less than 0.05 for either of the sample in each pair wise comparison were considered to be significantly differentially expressed.

### Quantitative real-time PCR (qRT-PCR)

Total RNAs were extracted using the Trizol (Invitrogen) following the protocols provided by the manufacturer. The DNA was digested with RNase-free DNase I (Promega). RNA of about 0.5 μg was reverse-transcribed into first-strand cDNA with PrimeScript® RT reagent Kit (TaKaRa). Quantitative real-time PCR (qRT-PCR) was conducted by ABI Stepone Plus instrument. Gene-specific and internal control primers were given in Supplementary Table S7. We performed three independent experiments from three biological repeats for qRT-PCR, and three measurements were made for each cDNA with an annealing temperature of 56 °C and a total of 40 cycles of amplification. The relative expression levels were calculated by the comparative C_T_ method.

### Gene ontology (GO) enrichment analysis

Analyses of Gene Ontology (GO) enrichment for differentially expressed genes or lncRNA target genes were performed by the GOseq R package, with correction of gene length bias [73]. GO terms with corrected *P* values less than 0.05 were taken as the significantly enriched by differentially expressed genes. We constructed interaction-networks among lncRNAs and protein-coding RNAs based on co-expression and genomic co-location by the software Cytoscape [74].

### Statistical analysis

All experiments in this study were repeated independently at least three times. The results are given means ± SE. The statistical analysis was performed using SPSS17.0 software (Chicago, IL, USA). The *t*-test was used to determine whether effects of treatments were statistically different at *p* < 0.05 level.

## Supplementary information

**Additional file 1: Table S1.** Statistical date of the RNA-Seq quality for experimental samples. **Table S2.** Correlation coefficient of lncRNAs for experimental samples. **Table S3.** Correlation coefficient of mRNAs for experimental samples. **Table S4.** Statistical date of the RNA-Seq results mapped to *M. truncatula* A17 genome for experimental samples. **Table S5.** GO enhancements of the putative targets of cold-responsive lncRNAs in leaves of *M. truncatula* seedlings. **Table S6.** GO enhancements of the putative targets of cold-responsive lncRNAs in roots of *M. truncatula* seedlings. **Table S7.** Primer sequences used for real-time quantitative RT-PCR.

**Additional file 2: Fig. S1.** Density distribution of lncRNAs on eight chromosomes of *M. truncatula* seedlings.

**Additional file 3: Fig. S2.** Number and density distribution of lncRNAs on chromosome of *M. truncatula* seedlings with and without cold treatment.

**Additional file 4: Fig. S3.** Identification of mRNAs by high-throughput sequencing in *M. truncatula* seedlings.

**Additional file 5: Fig. S4.** Characteristics of cold-responsive mRNAs in *M. truncatula* seedlings.

**Additional file 6: Fig. S5.** Protein sequence alignment of the MtCBFs and AtCBFs was constructed by MAGE.

**Additional file 7: Fig. S6.** Phylogenetic tree of MtCBFs and AtCBFs was constructed by MEGA.

**Additional file 8: Data Set S1.** lncRNA TPM result.

**Additional file 9: Data Set S2.** lncRNA sequence.

**Additional file 10: Data Set S3.** mRNA TPM result.

**Additional file 11: Data Set S4.** mRNA sequence.

## Data Availability

All data generated or analyzed during this study are included in this published article and its supplementary information files.

## Abbreviations

Antisense-lncRNA: LncRNA overlapping with reference on the opposite strand
*CAMTA*s: Calmodulin binding Transcription Activators
CAS: Cold-Acclimation-Specific
*CBF/DREB1*: C-Repeat Binding Factor/Dehydration-Responsive Element Binding factor 1
CNCI: Coding-Non-Coding-Index
COR: CRT/DRE-containing Cold-regulated
CPC: Coding Potential Calculator
CT: Cold Treated
DEG: Differentially expressed gene
FC(log_2_): Log2-transformed fold change
GO: Gene Ontology
IncRNA: Intergenic lncRNA
Intronic-lncRNA: lncRNA falling entirely within a reference intron
lncRNAs: Long noncoding RNAs
*MtCIR1*: *M. truncatula CBF* Intergenic RNA 1
NT: Non-cold Treated
ORFs: Open Reading Frames
PhyloCSF: Phylogenetic Codon Substitution Frequency
QC: Quality Control
*RDM4*: RNA-directed DNA methylation 4
RNA-seq: RNA sequencing
TPM: Transcript per kilobase of exon per million fragments mapped
WGCNA: Weighted Gene Co-expression Network Analysis
