# Supplementary material for: Identification of tissue-specific and cold-responsive lncRNAs in Medicago truncatula by high-throughput RNA sequencing
Source: BMC Plant Biol. 2020 Mar 6;20:99. doi: 10.1186/s12870-020-2301-1 (PMC7059299; doi:10.1186/s12870-020-2301-1)
Supplement: Supplementary file 1 — Additional file 1: Table S1. Statistical date of the RNA-Seq quality for experimental samples. Table S2. Correlation coefficient of lncRNAs for experimental samples. Table S3. Correlation coefficient of mRNAs for experimental samples. Table S4. Statistical date of the RNA-Seq results mapped to M. truncatula A17 genome for experimental samples. Table S5. GO enhancements of the putative targets of cold-responsive lncRNAs in leaves of M. truncatula seedlings. Table S6. GO enhancements of the putative targets of cold-responsive lncRNAs in roots of M. truncatula seedlings. Table S7. Primer sequences used for real-time quantitative RT-PCR. [file 12870_2020_2301_MOESM1_ESM.pdf]

Table S1. Statistical date of the RNA-Seq quality for experimental samples

Sample name	Error rate(%)	Q20(%)	Q30(%)	GC content(%)
NT_L_1	0.020	95.78	89.99	40.26
NT_L_2	0.020	95.51	89.45	40.36
NT_L_3	0.010	98.23	95.38	40.32
NT_R_1	0.020	96.58	91.65	40.67
NT_R_2	0.020	96.61	91.73	41.13
NT_R_3	0.020	96.79	92.10	40.82
CT_L_1	0.010	98.32	95.55	40.24
CT_L_2	0.010	98.25	95.41	40.18
CT_L_3	0.010	98.50	95.96	40.29
CT_R_1	0.020	96.53	91.55	40.72
CT_R_2	0.010	98.36	95.64	41.07
CT_R_3	0.020	96.45	91.38	40.70

Table 2. Correlation coefficient of lncRNAs for experimental samples

	NT_L_1	NT_L_2	NT_L_3	NT_R_1	NT_R_2	NT_R_3	CT_L_1	CT_L_2	CT_L_3	CT_R_1	CT_R_2	CT_R_3
**NT_L_1**	1.000	0.996	0.980	0.287	0.289	0.299	0.979	0.982	0.903	0.292	0.283	0.305
**NT_L_2**	0.996	1.000	0.969	0.258	0.259	0.269	0.974	0.975	0.884	0.266	0.253	0.278
**NT_L_3**	0.980	0.969	1.000	0.241	0.237	0.249	0.986	0.990	0.957	0.250	0.228	0.255
**NT_R_1**	0.287	0.258	0.241	1.000	0.954	0.978	0.289	0.280	0.277	0.948	0.907	0.969
**NT_R_2**	0.289	0.259	0.237	0.954	1.000	0.992	0.299	0.285	0.291	0.888	0.983	0.947
**NT_R_3**	0.299	0.269	0.249	0.978	0.992	1.000	0.307	0.2945	0.298	0.920	0.967	0.963
**CT_L_1**	0.979	0.974	0.986	0.289	0.299	0.307	1.000	0.998	0.963	0.294	0.297	0.308
**CT_L_2**	0.982	0.975	0.990	0.280	0.288	0.294	0.998	1.000	0.962	0.288	0.281	0.299
**CT_L_3**	0.903	0.884	0.957	0.277	0.291	0.298	0.963	0.962	1.000	0.279	0.289	0.291
**CT_R_1**	0.292	0.266	0.250	0.948	0.889	0.920	0.294	0.288	0.279	1.000	0.865	0.977
**CT_R_2**	0.283	0.253	0.228	0.907	0.983	0.967	0.297	0.281	0.289	0.865	1.000	0.935
**CT_R_3**	0.305	0.278	0.255	0.969	0.947	0.963	0.308	0.299	0.291	0.977	0.935	1.000

Table 3. Correlation coefficient of mRNAs for experimental samples

	NT-L-1	NT-L-2	NT-L-3	NT-R-1	NT-R-2	NT-R-3	CT-L-1	CT-L-2	CT-L-3	CT-R-1	CT-R-2	CT-R-3
**NT-L-1**	1.000	0.989	0.998	0.296	0.235	0.373	0.992	0.997	0.990	0.471	0.126	0.196
**NT-L-2**	0.989	1.000	0.981	0.301	0.241	0.373	0.995	0.991	0.990	0.468	0.137	0.206
**NT-L-3**	0.998	0.981	1.000	0.289	0.226	0.368	0.988	0.996	0.990	0.466	0.115	0.186
**NT-R-1**	0.296	0.301	0.289	1.000	0.979	0.988	0.308	0.300	0.304	0.955	0.951	0.982
**NT-R-2**	0.235	0.241	0.226	0.979	1.000	0.984	0.247	0.238	0.243	0.909	0.988	0.977
**NT-R-3**	0.373	0.373	0.368	0.988	0.984	1.000	0.384	0.377	0.381	0.952	0.952	0.968
**CT-L-1**	0.992	0.995	0.988	0.308	0.247	0.384	1.000	0.997	0.998	0.476	0.140	0.210
**CT-L-2**	0.997	0.991	0.996	0.300	0.238	0.377	0.997	1.000	0.997	0.473	0.129	0.199
**CT-L-3**	0.99	0.99	0.99	0.304	0.243	0.381	0.998	0.997	1.000	0.474	0.134	0.204
**CT-R-1**	0.471	0.468	0.466	0.955	0.909	0.952	0.476	0.473	0.474	1.000	0.866	0.933
**CT-R-2**	0.126	0.137	0.116	0.951	0.988	0.952	0.140	0.129	0.134	0.866	1.000	0.973
**CT-R-3**	0.196	0.206	0.186	0.982	0.977	0.968	0.210	0.199	0.204	0.933	0.973	1.000

Table S4. Statistical date of the RNA-Seq results mapped to *M. truncatula* A17 genome for experimental samples

Sample name	Clean reads	Total mapped	Multiple mapped	Uniquely mapped	Read-1	Read-2	Reads map to '+'	Reads map to '-'	Reads mapped in proper pairs
NT-L-1	92169948	86896605	30985337	55911268	28067200	27844068	27730194	28181074	53233478
		(94.28%)	(33.62%)	(60.66%)	(30.45%)	(30.21%)	(30.09%)	(30.58%)	(57.76%)
NT-L-2	93513730	87546360	29988076	57558284	28914856	28643428	28520097	29038187	54538276
		(93.62%)	(32.07%)	(61.55%)	(30.92%)	(30.63%)	(30.5%)	(31.05%)	(58.32%)
NT-L-3	113601816	110557503	42730774	67826729	34001540	33825189	33706568	34120161	65998606
		(97.32%)	(37.61%)	(59.71%)	(29.93%)	(29.78%)	(29.67%)	(30.03%)	(58.1%)
NT-R-1	88455680	80668827	4026406	76642421	38611417	38031004	38293230	38349191	73906842
		(91.2%)	(4.55%)	(86.64%)	(43.65%)	(42.99%)	(43.29%)	(43.35%)	(83.55%)
NT-R-2	88997680	81660066	4343486	77316580	38967067	38349513	38630026	38686554	74535908
		(91.76%)	(4.88%)	(86.87%)	(43.78%)	(43.09%)	(43.41%)	(43.47%)	(83.75%)
NT-R-3	90582052	83142057	4587164	78554893	39573527	38981366	39248933	39305960	75919764
		(91.79%)	(5.06%)	(86.72%)	(43.69%)	(43.03%)	(43.33%)	(43.39%)	(83.81%)
CT-L-1	108746292	105719232	37888424	67830808	33971375	33859433	33741435	34089373	65984844
		(97.22%)	(34.84%)	(62.38%)	(31.24%)	(31.14%)	(31.03%)	(31.35%)	(60.68%)
CT-L-2	105490866	102648368	38733525	63914843	32012318	31902525	31769188	32145655	62191732
		(97.31%)	(36.72%)	(60.59%)	(30.35%)	(30.24%)	(30.12%)	(30.47%)	(58.95%)
CT-L-3	103548450	100855019	38887409	61967610	31019886	30947724	30874036	31093574	60295986
		(97.4%)	(37.55%)	(59.84%)	(29.96%)	(29.89%)	(29.82%)	(30.03%)	(58.23%)
CT-R-1	86248354	79361542	4014056	75347486	37991326	37356160	37643816	37703670	72722254
		(92.02%)	(4.65%)	(87.36%)	(44.05%)	(43.31%)	(43.65%)	(43.72%)	(84.32%)
CT-R-2	93165358	88188250	4332172	83856078	42093610	41762468	41915420	41940658	82246364
		(94.66%)	(4.65%)	(90.01%)	(45.18%)	(44.83%)	(44.99%)	(45.02%)	(88.28%)
CT-R-3	86598790	79403338	3778624	75624714	38131904	37492810	37780962	37843752	72922078
		(91.69%)	(4.36%)	(87.33%)	(44.03%)	(43.29%)	(43.63%)	(43.7%)	(84.21%)

Table S5. GO enhancements of mRNAs co-expressed with lncRNAs in leaves of *M. truncatula* seedlings responding to cold treatment. The reliability is calculated by –$\log_{10}$ (*P*-value).

**GO Id**	**Function**	**GO term**	**-log10(*p*-value)**
GO:0006412	biological process	translation	12.61387
GO:0006352	biological process	DNA-templated transcription, initiation	4.179653
GO:0006486	biological process	protein glycosylation	4.036294
GO:0006886	biological process	intracellular protein transport	3.913072
GO:0006952	biological process	defense response	3.85942
GO:0006284	biological process	base-excision repair	3.258924
GO:0007018	biological process	microtubule-based movement	2.924919
GO:0016192	biological process	vesicle-mediated transport	2.538278
GO:0006520	biological process	cellular amino acid metabolic process	2.284524
GO:0016579	biological process	protein deubiquitination	2.207206
GO:0005622	cellular component	intracellular	15.60389
GO:0005840	cellular component	ribosome	12.05633
GO:0005737	cellular component	cytoplasm	6.091582
GO:0000786	cellular component	nucleosome	4.085975
GO:0005739	cellular component	mitochondrion	2.362878
GO:0031012	cellular component	extracellular matrix	2.155373
GO:0032040	cellular component	small-subunit processome	1.715167
GO:0005787	cellular component	signal peptidase complex	1.661857
GO:0009522	cellular component	photosystem I	1.529543
GO:0005634	cellular component	nucleus	1.373058
GO:0003735	molecular function	structural constituent of ribosome	13.31327
GO:0003676	molecular function	nucleic acid binding	10.68795
GO:0003755	molecular function	peptidyl-prolyl cis-trans isomerase activity	4.554987
GO:0003824	molecular function	catalytic activity	4.276863
GO:0008017	molecular function	microtubule binding	4.065523
GO:0016787	molecular function	hydrolase activity	3.694755
GO:0008378	molecular function	galactosyltransferase activity	2.980969
GO:0003777	molecular function	microtubule motor activity	2.924919
GO:0008270	molecular function	zinc ion binding	2.745694
GO:0005524	molecular function	ATP binding	2.725992

Table S6. GO enhancements of mRNAs co-expressed with lncRNAs in roots of *M. truncatula* seedlings responding to cold treatment. The reliability is calculated by –$\log_{10}$ (*P*-value).

**GO Id**	**Function**	**GO term**	**-log10(*p*-value)**
GO:0006412	biological process	translation	8.887366
GO:0006355	biological process	regulation of transcription, DNA-templated	6.140823
GO:0006351	biological process	transcription, DNA-templated	3.683584
GO:0006352	biological process	DNA-templated transcription, initiation	3.319818
GO:0006952	biological process	defense response	2.779378
GO:0006284	biological process	base-excision repair	2.77534
GO:0006396	biological process	RNA processing	2.695504
GO:0006886	biological process	intracellular protein transport	2.389622
GO:0042254	biological process	ribosome biogenesis	2.387814
GO:0006289	biological process	nucleotide-excision repair	2.337641
GO:0005622	cellular component	intracellular	8.899879
GO:0005737	cellular component	cytoplasm	8.858026
GO:0005840	cellular component	ribosome	8.209149
GO:0005634	cellular component	nucleus	2.592008
GO:0000786	cellular component	nucleosome	2.361815
GO:0005783	cellular component	endoplasmic reticulum	2.140334
GO:0016592	cellular component	mediator complex	1.874238
GO:0005741	cellular component	mitochondrial outer membrane	1.68588
GO:0005789	cellular component	endoplasmic reticulum membrane	1.593837
GO:0005730	cellular component	nucleolus	1.486506
GO:0003735	molecular function	structural constituent of ribosome	9.121137
GO:0003676	molecular function	nucleic acid binding	8.598387
GO:0003700	molecular function	DNA binding transcription factor activity	6.985659
GO:0005525	molecular function	GTP binding	4.695768
GO:0003924	molecular function	GTPase activity	4.129377
GO:0005509	molecular function	calcium ion binding	3.339094
GO:0008017	molecular function	microtubule binding	3.151118
GO:0016787	molecular function	hydrolase activity	2.954905
GO:0051082	molecular function	unfolded protein binding	2.605583
GO:0003723	molecular function	RNA binding	2.451817

Table S7. Primer sequences used for real-time quantitative RT-PCR

MtACTIN (*Mt7g026230*)-F	ACGAGCGTTTCAGATG
MtACTIN (*Mt7g026230*)-R	ACCTCCGATCCAGACA
MtCIR1-F	CTTACGTTACTGCCCAAGTGAAA
MtCIR1-R	GCAGCTCCTTCGTACAAGACAT
Mt6g465420-F	TGATGAACAGGAGGAAA
Mt6g465420-R	AAAGCGGGGTTCGCAGTCAC
Mt6g465430-F	GTTGTAGCTGTTGTCGCAACACAGG
Mt6g465430-R	TCAGACACATAAAAGCAAGGTTCGC
Mt6g465450-F	TTCGGAGGTACGATTAGCGGCTAGT
Mt6g465450-R	TAGGAAGGCGCCACACCGAGTCTGC
Mt6g465460-F	GAGTTTTGGAGGAATATGGCACTAA
Mt6g465460-R	TCAAGTTTCATTCATATTCTTCTTT
Mt6g465510-F	TCACAGAAGAACTTAGTTTATTTTG
Mt6g465510-R	TAGAAGTTCCATAGGGATACCTCTT
Mt6g465530-F	ATGTATACAAGTAACAACGGGGA
Mt6g465530-R	GTAGCGGCCCCTCAATGCAAT
Mt6g465690-F	GAAACTTAGATAAATGGGTG
Mt6g465690-R	GTAGCAGAGGTGGAAATAG
