# Supplementary material for: Identification of tissue-specific and cold-responsive lncRNAs in Medicago truncatula by high-throughput RNA sequencing
Source: BMC Plant Biol. 2020 Mar 6;20:99. doi: 10.1186/s12870-020-2301-1 (PMC7059299; doi:10.1186/s12870-020-2301-1)
Supplement: Supplementary file 2 — Additional file 2: Fig. S1. Density distribution of lncRNAs on eight chromosomes of M. truncatula seedlings. [file 12870_2020_2301_MOESM2_ESM.pdf]

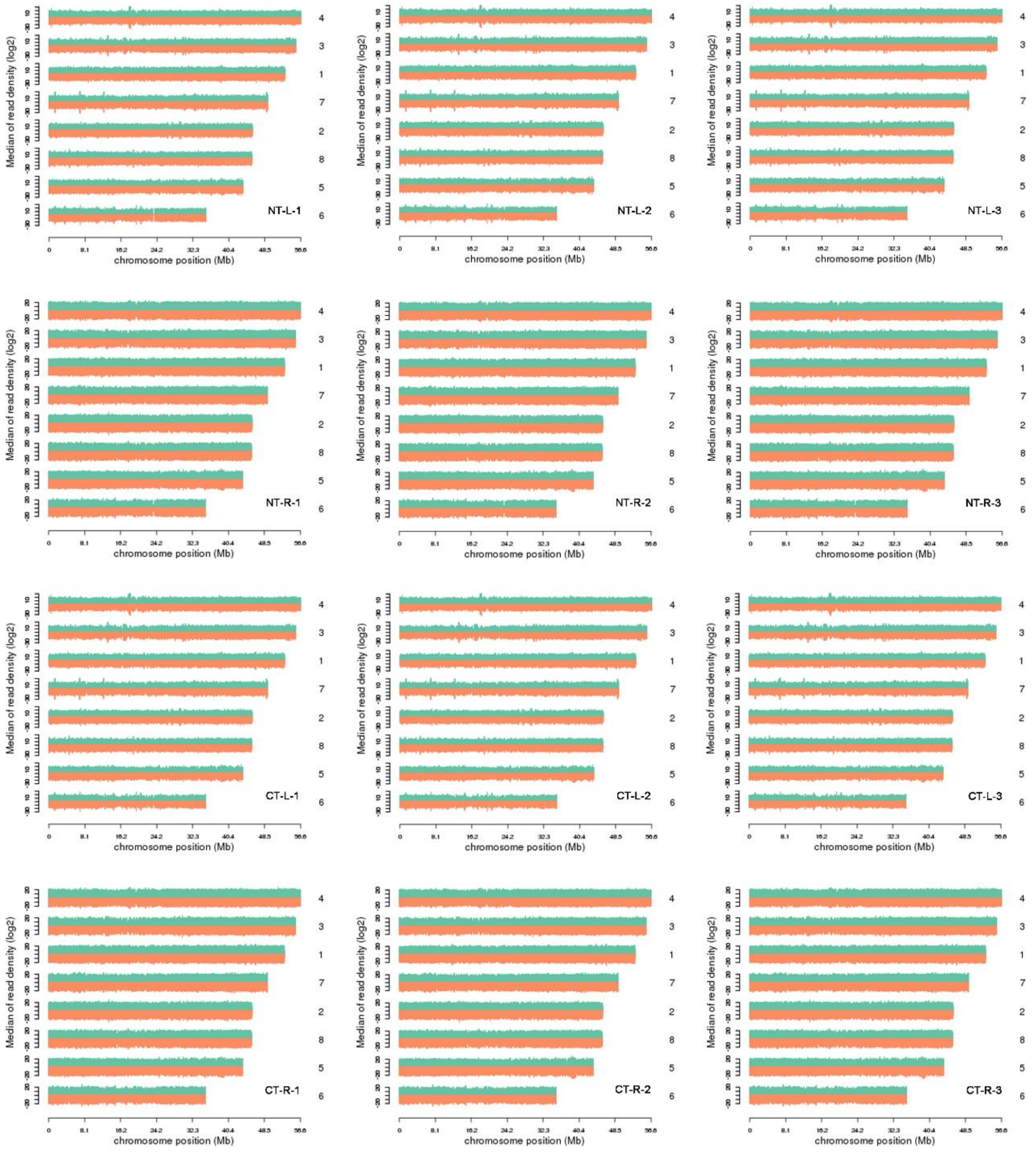

Figure S1. Density distribution of lncRNAs on eight chromosomes of *M. truncatula* seedlings with three biological repeats for four treatments of NT-L, NT-R, CT-L, CT-R. NT (non-cold acclimated). CT (cold acclimated). L (leaves). R (roots).
