# Supplementary material for: Identification of tissue-specific and cold-responsive lncRNAs in Medicago truncatula by high-throughput RNA sequencing
Source: BMC Plant Biol. 2020 Mar 6;20:99. doi: 10.1186/s12870-020-2301-1 (PMC7059299; doi:10.1186/s12870-020-2301-1)
Supplement: Supplementary file 3 — Additional file 3: Fig. S2. Number and density distribution of lncRNAs on chromosome of M. truncatula seedlings with and without cold treatment. [file 12870_2020_2301_MOESM3_ESM.pdf]

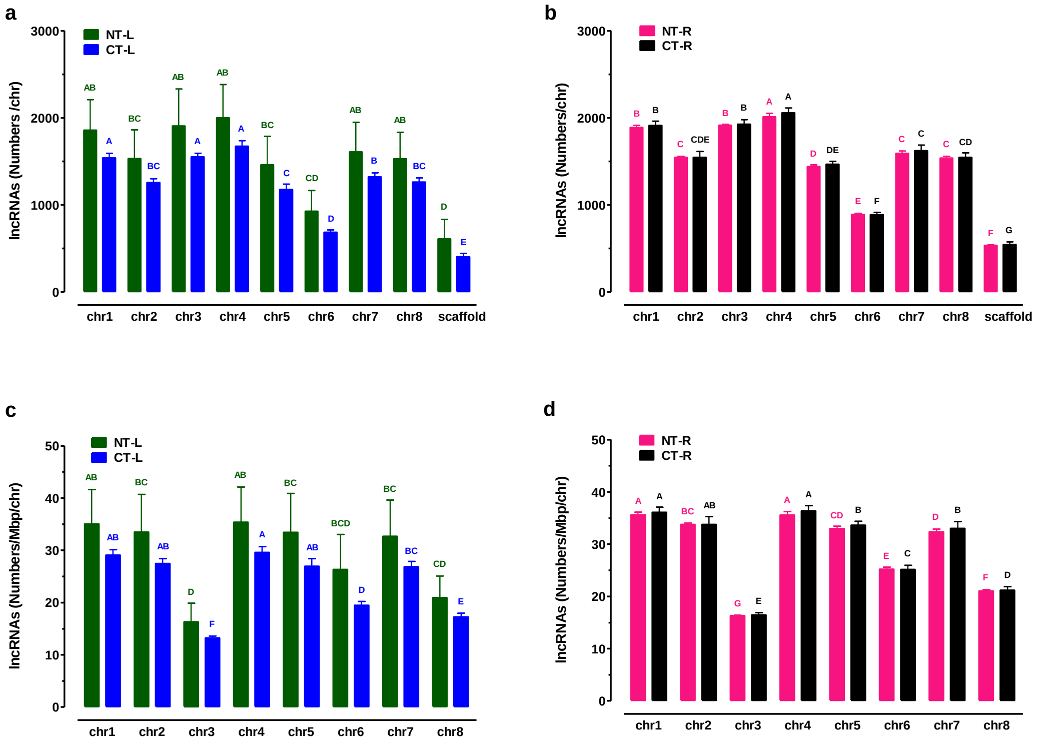

Figure S2. Number and density distribution of lncRNAs on chromosome of *M. truncatula* seedlings with and without cold treatment. Number distribution of lncRNAs on chromosomes of leaves (**a**) and roots (**b**) with and without cold treatment. Density distribution of lncRNAs on chromosomes of leaves (**c**) and roots (**d**) with and without cold treatment. Values are means ± SE with three independent RNAseq experiments. Capital letters indicate a significant different at P<0.05 according to t-test between chromosomes in same treatment samples. NT: non-cold treated; CT: cold treated; L: leaves; R: roots.
