# Supplementary material for: Identification of tissue-specific and cold-responsive lncRNAs in Medicago truncatula by high-throughput RNA sequencing
Source: BMC Plant Biol. 2020 Mar 6;20:99. doi: 10.1186/s12870-020-2301-1 (PMC7059299; doi:10.1186/s12870-020-2301-1)
Supplement: Supplementary file 4 — Additional file 4: Fig. S3. Identification of mRNAs by high-throughput sequencing in M. truncatula seedlings. [file 12870_2020_2301_MOESM4_ESM.pdf]

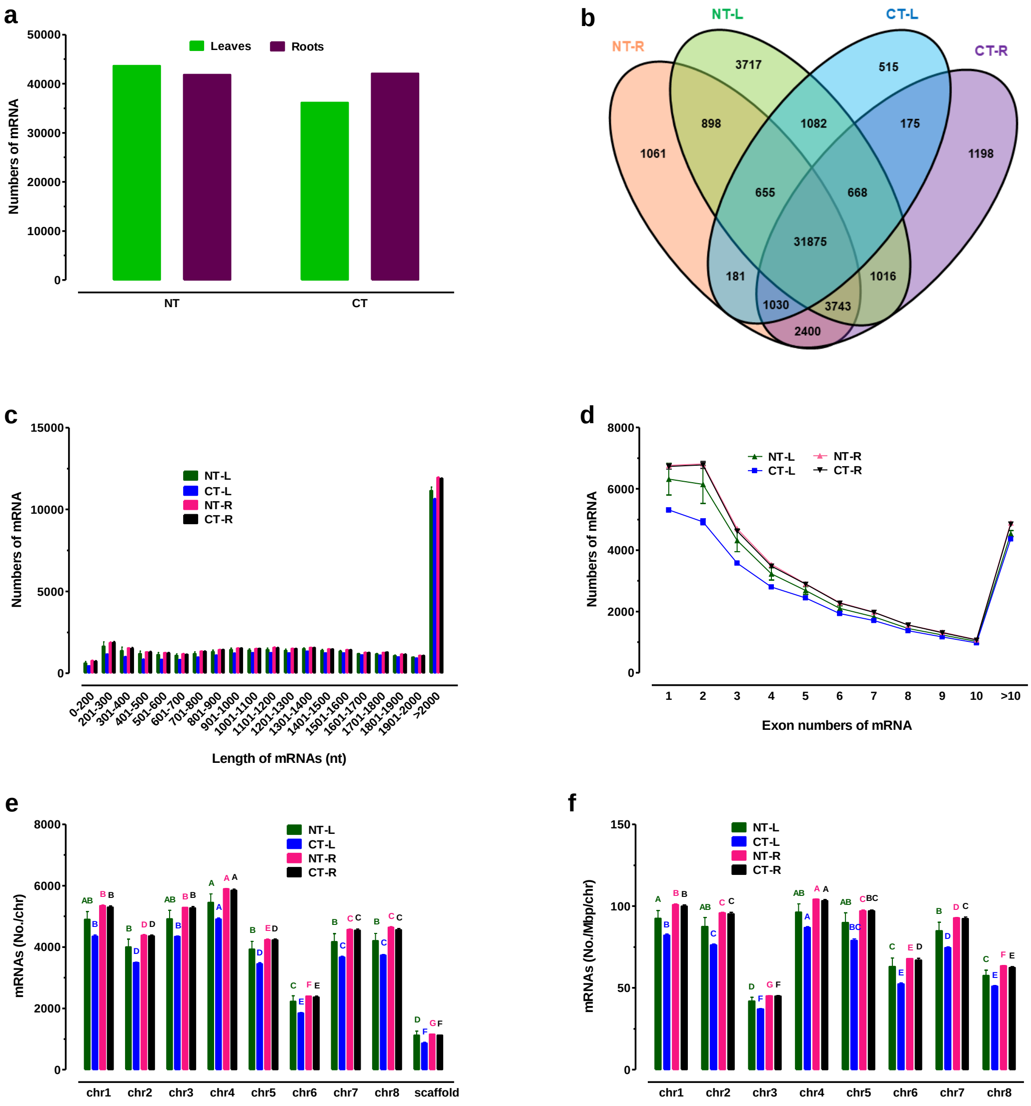

Figure S3. Identification of mRNAs by high-throughput sequencing in *M. truncatula* seedlings. (**a**) Total numbers of mRNA identified in leaves and roots. (**b**) Numbers of common/specific mRNA identified in NT-L, CT-L, NT-R and CT-R. The values shown in (**a**) and (**b**) were obtained from three independent RNAseq experiments. (**c**) Length distribution of mRNAs in leaves and roots. (**d**) Numbers of mRNA containing different numbers of exon in NT-L, CT-L, NT-R and CT-R. (**e**) Number distribution of mRNAs on eight chromosomes of leaves and roots with and without cold treatment. (**f**) Density distribution of mRNAs on eight chromosomes of leaves and roots with and without cold treatment. Capital letters indicate significant difference at $P<0.05$ according to the *t*-test between chromosomes in the same treatment samples. Values

shown in (**c**) (**d**) (**e**) and (**f**) were means ± SE with three independent RNAseq experiments. NT: non-cold treated; CT: cold treated; L: leaves; R: roots.
