# Supplementary material for: Identification of tissue-specific and cold-responsive lncRNAs in Medicago truncatula by high-throughput RNA sequencing
Source: BMC Plant Biol. 2020 Mar 6;20:99. doi: 10.1186/s12870-020-2301-1 (PMC7059299; doi:10.1186/s12870-020-2301-1)
Supplement: Supplementary file 5 — Additional file 5: Fig. S4. Characteristics of cold-responsive mRNAs in M. truncatula seedlings. [file 12870_2020_2301_MOESM5_ESM.pdf]

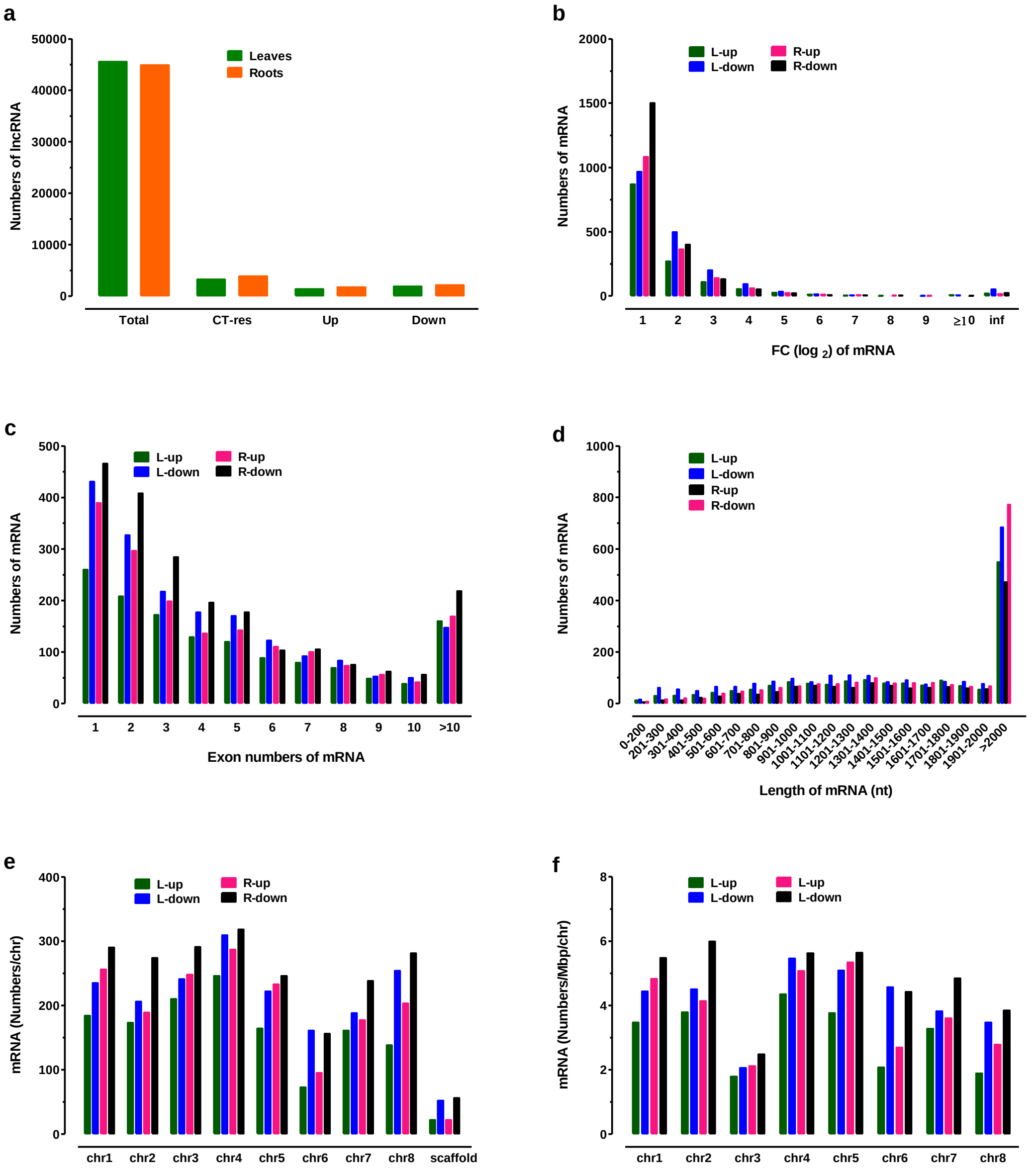

Figure S4. Characteristics of cold-responsive mRNAs in *M. truncatula* seedlings. (**a**) Numbers of cold-responsive mRNAs in leaves and roots of *M. truncatula* seedlings. (**b**) Numbers of mRNAs with different FC(log_2) which were regulated by cold treatment in leaves and roots of *M. truncatula* seedlings. Data on X axis indicated varying (FC(log_2)) values ranging from ⩾1 to =10. (**c**) Numbers of mRNAs containing different exon numbers in response to cold treatment in leaves and roots of *M. truncatula* seedlings. (**d**) Numbers of mRNAs with different length responded to cold treatment in leaves and roots of *M. truncatula* seedlings. (**e**) Numbers of mRNAs distributed on chromosomes in response to cold treatment in leaves and roots of *M.*

truncatula seedlings. (**f**) Densities of mRNAs on eight chromosomes responded to cold treatment in leaves and roots of *M. truncatula* seedlings. CT-res: cold responsive-mRNAs; up- or down-regulated: expression of mRNAs induced or reduced by cold treatment; L: leaves; R: roots.
