# Supplementary material for: Identification of tissue-specific and cold-responsive lncRNAs in Medicago truncatula by high-throughput RNA sequencing
Source: BMC Plant Biol. 2020 Mar 6;20:99. doi: 10.1186/s12870-020-2301-1 (PMC7059299; doi:10.1186/s12870-020-2301-1)
Supplement: Supplementary file 6 — Additional file 6: Fig. S5. Protein sequence alignment of the MtCBFs and AtCBFs was constructed by MAGE. [file 12870_2020_2301_MOESM6_ESM.pdf]

```
At4g25470      --MNSFSAFS EMFG------ ---------- SDYESPVSSG GDYSPKLATS C-PKKPAGRK
At4g25480      --MNSFSAFS EMFG------ ---------- SDYESSVSSG GDYIPTLASS C-PKKPAGRK
At4g25490      --MNSFSAFS EMFG------ ---------- SDYEP---QG GDYCPTLATS C-PKKPAGRK
Mt1g101600     --MN------ ---------- ---------- QFSESHDPCS SSSERFLAET M-PKKRAGRK
Mt4g102660     ---MKSSLDE SSYVE----- ---------- NNS------- SSSEILLASE Q-PKKRAGRR
Mt5g010910     MDFFMSSFGE YSDTS----- ---------- SSE------T SSSEVILASV NKPKKRAGRR
Mt5g010930     MNIFLSSFGD YSDSS----- ---------- SSS-----ET SSSEVILASA R-PKKRAGRR
Mt5g010940     MDFFMSSFSD YSDTS----- ---------- SSETASSNRT SSSEVILAPA R-PKKRAGRR
Mt6g465420     --MISTNNSS YSHSISS--- ---------- KDFSPFDASS PGSEVRLASS N-PKKRAGRK
Mt6g465430     --MISTNNSS YSHSISS--- ---------- KDFSPFDASS PDSEVRLASS N-PKKRAGRK
Mt6g465450     --MFTTNNSS YSQSIPS--- ---------- TASSSYDMST PNSEVRLAAS N-PKKRAGRK
Mt6g465460     --MFTTNNSS YSHSISS--- ---------- EASSSYYNSL PESEIRLAAS N-PKKRAGRK
Mt6g465510     --MFTNNNG- ---------- ---------- ---------- ---EGRLAES C-PKKPAGRK
Mt6g465530     --MYTSNNG- ---------- ---------- ---------- ---EGRLAAS C-PKKPAGRK
Mt6g465690     MDMFTNNNSY SHPFSPT--- --CSESS-FP NSEGSQGMSI SNEEVRLAAT T-PKKRAGRK
Mt6g465850     --MFTTNNSI YSHPSSS--- SLSHIIDEFR GLSYHR-MSV SNKEVRLAVT T-PKKRGGRK
Mt6g465990     ---------- ---------- -----MS-LP NSGGSHWMSI CNEEMRLAAT T-PKKRAGRK
Mt6g466000     --MYPTTN-- --SVSSS--- --SSDMS-LP NSEGSHWMSI CNEEMRLAAT T-PKKRAGRK
Mt6g466020     ---------- ---------- -----MS-LP NLEVSHGTPI STEDVRLAAS T-PKKRACRK
Mt6g466130     --MFPSNNPS SSHPSSSKPS SSSHTIS-LP NLEVSRGMFV TNDEVRLAST T-PKKRAGRK

At4g25470      KFRETRHPIY RGVRQRNSG- -KWVCELREP N------KKT RIWLGTFQTA EMAARAHDVA
At4g25480      KFRETRHPIY RGVRRRNSG- -KWVCEVREP N------KKT RIWLGTFQTA EMAARAHDVA
At4g25490      KFRETRHPIY RGVRQRNSG- -KWVSEVREP N------KKT RIWLGTFQTA EMAARAHDVA
Mt1g101600     KFRETRHPVY RGVRKRDSG- -KWVCEVREP N------KKT RIWLGTFPTP EMAARAHDVA
Mt4g102660     KFKETRHPVY RGVRRRNNNN NKWVCEVRVP N-----DKST RIWLGTYPTP EMAARAHDVA
Mt5g010910     VFKETRHPVY RGVRRRKNN- -KWVCEMRVP NNIVNKNNKS RIWLGTYPTP EMAARAHDVA
Mt5g010930     VFKETRHPVY RGVRRRNNN- -KWVCEMRVP NINVNKNNKS RIWLGTYPTP EMAARAHDVA
Mt5g010940     VFKETRHPVY RGVRRRKNN- -KWVCEMRVP NNIVNKNNKS RIWLGTYPTP EMAARAHDVA
Mt6g465420     IFKETRHPVY RGVRKRNLN- -KWVCEMREP N------TKN RIWLGTFPTP EMAARAHDVA
Mt6g465430     IFKETRHPVY RGVRKRNLN- -KWVCEMREP N------TKN RIWLGTFPTA EMAARAHDVA
Mt6g465450     IFKETRHPMY RGVRKRNLD- -KWVCEMREP N------TKT RIWLGTFPTP EMAARAHDVA
Mt6g465460     IFKETRHPVY RGVRKRNLD- -KWVCEMREP N------TKT RIWLGTFPTA EMAARAHDVA
Mt6g465510     KFKETRHPVY RGVRKRNLD- -KWVCEMREP N------KKT RIWLGTFPTA EMAARAHDVA
Mt6g465530     KFKETRHPVY RGVRKRNLD- -KWVCEMREP N------KKT RIWLGTFATA EMAARAHDVA
Mt6g465690     KFKETRHPVY RGVRKRNLD- -KWVCEMREP N------KKT KIWLGTFPTA EMAARAHDVA
Mt6g465850     KFKETRHPVY RGVRSRNLD- -KWVCEIRQP N------KKT KIWLGTFPTA EMAARAHDVA
Mt6g465990     KFKETRHPVY RGVRKRNLD- -KWVCEMRKP N------KKT KIWLGTFPTA EMAT------
Mt6g466000     KFKETRHPVY RGVRKRNLD- -KWVCEMREP N------KKT KIWLGTFPTA EMAARAHDVA
Mt6g466020     KFKVTCHPIY RGVRSRNLG- -KWVCEMREP N------KMT KIWLGTFPMA EMEARAHDVA
Mt6g466130     KFKETRHPVY RGVRRRNLD- -KWVCEMREP N------KKT KIWLGTFPTA EMAARAHDVA

At4g25470      AIALRG---- ---------- ---------- ---------- ---------- ----------
At4g25480      ALALRG---- ---------- ---------- ---------- ---------- ----------
At4g25490      ALALRG---- ---------- ---------- ---------- ---------- ----------
Mt1g101600     AIALRG---- ---------- ---------- ---------- ---------- ----------
Mt4g102660     ALALRG---- ---------- ---------- ---------- ---------- ----------
Mt5g010910     ALTLKG---- ---------- ---------- ---------- ---------- ----------
Mt5g010930     ALTLKG---- ---------- ---------- ---------- ---------- ----------
Mt5g010940     ALTLKG---- ---------- ---------- ---------- ---------- ----------
Mt6g465420     AMALRG---- ---------- ---------- ---------- ---------- ----------
Mt6g465430     AIALRG---- ---------- ---------- ---------- ---------- ----------
Mt6g465450     AMALRG---- ---------- ---------- ---------- ---------- ----------
Mt6g465460     AMALRG---- ---------- ---------- ---------- ---------- ----------
Mt6g465510     AMALRG---- ---------- ---------- ---------- ---------- ----------
Mt6g465530     AIALRG---- ---------- ---------- ---------- ---------- ----------
Mt6g465690     AMALRG---- ---------- ---------- ---------- ---------- ----------
Mt6g465850     ALALKGGDAC LNFADSALTL PIPATCETKD IQKTAAEAAE AFRPGKTVMT NDVAALALRG
Mt6g465990     ---------- ---------- ---------- ---------- ---------- ----------
Mt6g466000     AMALRG---- ---------- ---------- ---------- ---------- ----------
Mt6g466020     TLALRG---- ---------- ---------- ---------- ---------- ----------
Mt6g466130     ALALRG---- ---------- ---------- ---------- ---------- ----------
```

```
At4g25470     RSACLNFADS AWRLRIPEST CAKEIQKAAA EAALNFQDEM CHMTTDA--- -HGLDMEETL
At4g25480     RSACLNFADS AWRLRIPEST CAKDIQKAAA EAALAFQDEM CDATTD---- -HGFDMEETL
At4g25490     RSACLNFADS AWRLRIPEST CAKDIQKAAA EAALAFQDET CDTTTTN--- -HGLDMEETM
Mt1g101600    RSACLNFADS AWKLPVPATS EARDIQKAAA EAAEAFRPES VFENS----- -----EERKD
Mt4g102660    KSACLNFADS AWRLALPATN NAKEIRKMAA EAALAFAVVA DSKEQTM--- -----ISNCD
Mt5g010910    KSACLNFADS AWRLRLPESN DATEIRRAAM EAAQLFAVED KQCCVTV--- -----EDGVF
Mt5g010930    KSACLNFADS AWRLTLPESN DAVEIRRAAM EAAKMFAIEE NHNQRSD--- -----RDAVD
Mt5g010940    KSACLNFADS AWRLRLPESN DATEIRRAAM EAAQLFAVED KQCCVTV--- -----EDGVF
Mt6g465420    RYACLNFSDS VWRLPIPATS AIKDIQKAAT KAAEAFRPDN TLMTN----- -----NIDTI
Mt6g465430    RYACLNFADS VWRLPIPATS AIKDIQKAAT KAAEAFRPDN TLMTS----- -----DIDTV
Mt6g465450    RYACLNFADS VWRLPIPATS AIKDIQKAAA EAAEAFRPDK TLMIN----- -----DIDTV
Mt6g465460    RYACLNFADS VWRLPIPATS SIKDIQKAAT KAAEAFRPDN TIMIT----- -----NIETV
Mt6g465510    RYACLNFADS VWRLPIPASA EAKDIQRAAA EAAEAFRPDK TLMTN----- -----DIDTV
Mt6g465530    RYACLNFADS AWKLPIPASA EAKDIQRAAG EAAEAFRPDK TLMTT----- -----GIDTV
Mt6g465690    RYACLNFADS AWRLPIPATT QAKDIQKAAA QAAEAFRPDK TSITN----- -----DIDTA
Mt6g465850    RNACLNFADS ASRLPIPATK ETRDIQNAAA EATEAFRPDK TLTTN----- -----DNETA
Mt6g465990    --------HS AWRLPKPATT QAKDIQKAAA EAAKAFRPDK TLLTNHNDND NDNDKENDMA
Mt6g466000    RYACLNFADS AWRLPKPATT QAKDIQKAAT EAAEAFRPDK TLLTNHNDND NDNDKENDMA
Mt6g466020    CYACLNFADS AWRLLIPATI KT-------- -SRGCHRGG- ---------- ----------
Mt6g466130    RNACLNFADS ASRLPIPATT ETRDIQKTAA EAAEAFRPDK TLTTD----- -----DNDNA

At4g25470     VEAIYTP--- ---------E QSQDAFYMDE EA----MLGM SSLLDNMAEG MLLPSPSVQW
At4g25480     VEAIYTA--- ---------E QSENAFYMHD EA----MFEM PSLLANMAEG MLLPLPSVQW
At4g25490     VEAIYTP--- ---------E QSEGAFYMDE ET----MFGM PTLLDNMAEG MLLPPPSVQW
Mt1g101600    SEPSST---- ---------- VAVSETVMEQ RE--EEEDTV PEYLRNMV-- LMSPAHYWGS
Mt4g102660    VNSVGVMEV- --------DN KPLQGLCVEV PEE-EMLHDW FRSMADEP-- LRSPITPFIR
Mt5g010910    MD-MEDSK-- --------NM LEAQVPVVSS EFE-DMHH-L LLSIANEP-- LRSAPPSPTN
Mt5g010930    MN-MENSKK- --------NV LEVQVPVLSS EFE-DMHHNL LLSIANEP-- LRSNPPSPTN
Mt5g010940    MD-MEDSK-- --------NM LEAQVPVVSS EFE-DMHH-L LLSIANEP-- LRSAPPSPTN
Mt6g465420    VDVVAT---- ---------- KELNMFCVEV DEQ-EEMLNM PELWRNMA-- LMSPTHSFEY
Mt6g465430    VAVVAT---- ---------- QELNMFRVEV EE--EEVLNM PELWRNMA-- LMSPTHSFGY
Mt6g465450    VPVVAT---- ---------- KELNMFCVEV EEE-QEMLNM PELLRNMA-- LMSPTHSFEY
Mt6g465460    VAVVAT---- ---------- KELNMFCVEE EEE---MLNM PEFWRNMA-- LMSPTHSFEY
Mt6g465510    VAVVVTEELS LFCVELEEEE EVLNMFCVEV EKE-EEVLNM SEMWRNMA-- LMSPTHSMEH
Mt6g465530    VAVVV----- ----AVEEEE EVLNMFCVEV EKE-EEVLNM QELWKNMA-- LMSPTHSFEH
Mt6g465690    ISTSAT---- ---------- AEQSRTFMEE EE--EGVMNM PELLRNMA-- LMSPTHSSGY
Mt6g465850    VAAAAA---- ---------- EEQLMFSMEE EEEEEEELNI PESLRNMV-- LMSPTHSLGH
Mt6g465990    VVATAT---- ---------- EEQSMICMEE KE--EGVMNM QEMWSNMA-- LMSPTHSLGY
Mt6g466000    VVATAT---- ---------- EEQSMICMEE KE--EGVMNM QEMWSNMA-- LMSPTHSLGY
Mt6g466020    -AYVSH---- ---------- HGKVFFGEEE EEKEEEVLNI PESLRNMA-- LMSPTHSVEH
Mt6g466130    VAAAAA---- ---------- EEQLMFSMEE EEEEEEELNI PESLRNMA-- LMSPTHSLGH

At4g25470     N---YNFDVE G-------DD D--------- ---------- -VSLWSY--- ----------
At4g25480     N---HNHEVD G-------DD DD-------- ---------- -VSLWSY--- ----------
At4g25490     N---HNYDGE G-------DG D--------- ---------- -VSLWSY--- ----------
Mt1g101600    DCGVADVE-- -------FDE T--------- ---------- EVSLWSYSF- ----------
Mt4g102660    HGRDQWNNVD I------DQV D--------- ---------A EVSLWNFTI- ----------
Mt5g010910    YGSYNWGDME I------FDT ---------- ---------Q LVSLWNFSI- ----------
Mt5g010930    YYGSNYDDME I------FDT ---------- ---------Q IVSLWNFSI- ----------
Mt5g010940    YGSYNWGDME I------FDT ---------- ---------Q LVSLWNFSI- ----------
Mt6g465420    H-EYDDIHVQ D------FQD DEDLKKKS-- ---------- VTTTWTVTAT GVHSPHFTIM
Mt6g465430    HDQYEDIHIQ D------FQD DEDFKKRS-- ---------- VTTIWAVTSI GVHSLHFTVI
Mt6g465450    HDQYEDIHVQ D------FQD DEDFKKKS-- ---------- VTTIWAVTAI GVHTPHFTVI
Mt6g465460    HDQYEDFHFQ D------FQD DEDFKRRS-- ---------- ITMSWVLTAI GVLSPHFTVT
Mt6g465510    E--YENFDLQ D------FQD EE-------- ---------- -VSLWNF--- ----------
Mt6g465530    E--YEDFDVQ -------FQD EE-------- ---------- -VSLWNF--- ----------
Mt6g465690    N-EYENIHVQ DFQDLQDFQD EEVLIKHKVL LIPSISIYER RIEVWYVKIS VNFISYLN--
Mt6g465850    --EFEHIDHG ------DFQD D--------- ---------- EVSLWSFTI- ----------
Mt6g465990    Y-EYQYIN-- -----EDFQD E--------- ---------- KVSLWSF--- ----------
Mt6g466000    Y-EYQYIN-- -----EDFQD E--------- ---------- KVSLWSF--- ----------
Mt6g466020    --EYQGIDA- ------DFQD I--------- ---------- EVSL------ ----------
Mt6g466130    --EFEHIDHG ------DFQD D--------- ---------- EVSLWSFSV- ----------
```

```
At4g25470     ---------- ---------- ---------- ------
At4g25480     ---------- ---------- ---------- ------
At4g25490     ---------- ---------- ---------- ------
Mt1g101600    ---------- ---------- ---------- ------
Mt4g102660    ---------- ---------- ---------- ------
Mt5g010910    ---------- ---------- ---------- ------
Mt5g010930    ---------- ---------- ---------- ------
Mt5g010940    ---------- ---------- ---------- ------
Mt6g465420    YRIVIVAVTA NPAFIF--SL FSSWQRKVEK KKE---
Mt6g465430    SRIVMRTLLL CV-------- ---------- ------
Mt6g465450    SRIVIVSM-- ---------- ---------- ------
Mt6g465460    SRIVIVAVTA NPTFLFLVSL FSSWQGKLQK KNMNET
Mt6g465510    ---------- ---------- ---------- ------
Mt6g465530    ---------- ---------- ---------- ------
Mt6g465690    ---------- ---------- ---------- ------
Mt6g465850    ---------- ---------- ---------- ------
Mt6g465990    ---------- ---------- ---------- ------
Mt6g466000    ---------- ---------- ---------- ------
Mt6g466020    ---------- ---------- ---------- ------
Mt6g466130    ---------- ---------- ---------- ------
```

Figure S4. Protein sequence alignment of the MtCBFs and AtCBFs was constructed by MAGE.
