# Supplementary figures and images for: Identification of tissue-specific and cold-responsive lncRNAs in Medicago truncatula by high-throughput RNA sequencing
Source: BMC Plant Biol. 2020 Mar 6;20:99. doi: 10.1186/s12870-020-2301-1 (PMC7059299; doi:10.1186/s12870-020-2301-1)

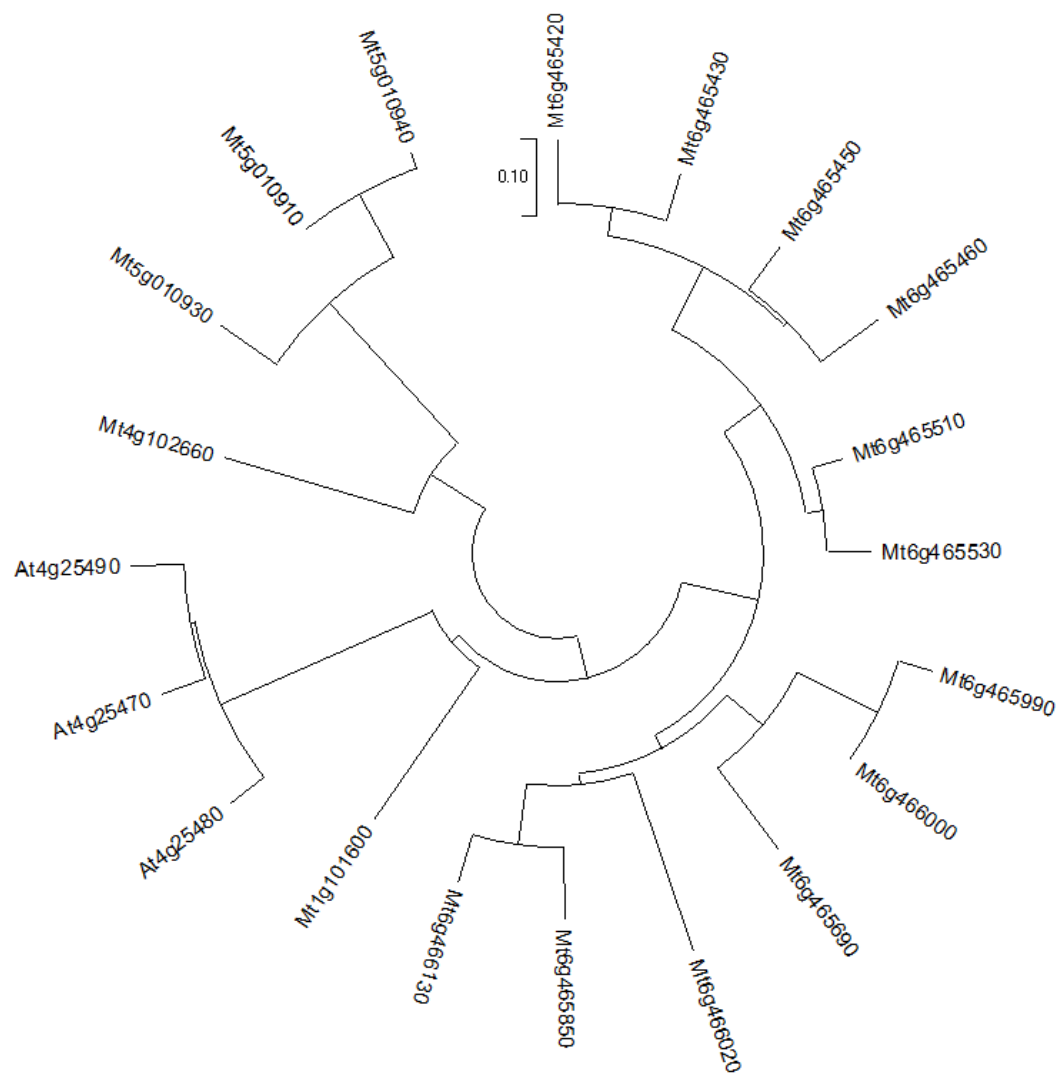

**Figure S6.** Phylogenetic tree of MtCBFs and AtCBFs was constructed by MEGA.

Supplement: Supplementary file 7 — Additional file 7: Fig. S6. Phylogenetic tree of MtCBFs and AtCBFs was constructed by MEGA. [file 12870_2020_2301_MOESM7_ESM.pdf]
